# CD271 orchestrates skin structure, differentiation, and inflammation via PI3K/Akt and PKCα/ERK pathways

**DOI:** 10.1038/s41419-025-08062-5

**Published:** 2025-10-21

**Authors:** Marika Quadri, Luca Reggiani Bonetti, Cristina Pellegrini, Mirco Mastrangelo, Andrea Caporali, Cristina Vaschieri, Roberta Lotti, Maria Concetta Fargnoli, Carlo Pincelli, Alessandra Marconi, Elisabetta Palazzo

**Affiliations:** 1 DermoLAB, Department of Surgical, Medical, Dental and Morphological Science, University of Modena and Reggio Emilia, Modena, Italy; 2 Department of Diagnostic, Clinic and Public Health Medicine, University of Modena and Reggio Emilia, Modena, Italy; 3 Department of Biotechnology and Applied Clinical Sciences, UnivAQ, Italy; 4 Centre for Cardiovascular Science, The University of Edinburgh, Edinburgh, UK; 5 San Gallicano Dermatological Institute, IRCCS, Rome, Italy

**Keywords:** Cell signalling, Experimental models of disease

## Abstract

The involvement of the neurotrophin network in pathological skin conditions, such as psoriasis or squamous cancer, by their common neurotrophin receptor CD271 has become recently evident. Depending on the specific ligand and co-receptor interacting with it, CD271 mediates various cellular responses in keratinocytes. In vitro analysis shows that it is implicated in the transition from human interfollicular keratinocyte stem cells to transient amplifying cells. However, no in vivo models are available to dissect the complexity of these mechanisms, including the effect on the inflammatory response. Here, we develop and characterize two novel mouse models, the CD271cKO and the CD271ciKO, where CD271 is conditionally absent in keratinocytes during development or after topical induction, respectively. By histology, functional assay, transcriptomics and molecular analysis, we identified substantial skin changes correlated to CD271 deletion, including epidermal hyperproliferation, “activated” keratinocyte signature, and a delayed in the differentiation process, mostly linked to PI3K/Akt and mitogenic pathways-dependent processes. KO keratinocyte displays upregulation of Ki67, PCNA, KRT5, KRT6, and ERK phosphorylation, as well as major expression of IL1α, Cxcl15, and TGFβ. KO skin resemble dysplastic skin conditions, including the recruitment of immune cells, particularly T cells, macrophages, and neutrophils, and release of inflammatory cytokines involved in TNF, JAK/Stat, IL17, and PI3k/Akt signaling pathways. Overall, our data defines CD271 as a crucial regulator of skin homeostasis. Therefore, our models represent an exceptionally useful tool for the characterization of skin pathophysiology linked to CD271 and possibly for developing appropriate therapies.

## Introduction

Neurotrophins (NTs) are growth factors that play crucial roles in both neuronal and non-neuronal tissues [1–4]. An increasing number of studies demonstrate their functional implications in skin physiology, where they influence several processes [4]. Human interfollicular keratinocyte (HIK) differentiation is a well-controlled process that involves the maturation of keratinocyte stem cells (KSCs) into transit amplifying (TA) cells that in turn determine the functional epithelium. Recently, a critical role of the common neurotrophin receptor CD271 in the initial stages of interfollicular KSC differentiation has been demonstrated, highlighting its impact on the KSC transition towards TA phenotype [5]. CD271 is highly expressed in an early TA cell population (ETA), progenitors with proliferative ability together with initial commitment [5, 6]. Accordingly, calcium-induced CD271 expression is associated with differentiation marker expression of HIK [7].

NT-dependent signaling has been also implicated in inflammatory skin diseases, such as alopecia, psoriasis, and atopic dermatitis, where NTs can contribute to cell loss or hyperproliferation [7–9]. Specifically, CD271 plays key roles within the skin pathological microenvironment characterized by altered cutaneous homeostasis [4]. In psoriasis, a decrease in CD271 levels, particularly in lesional skin, leads to apoptosis resistance and disrupted skin maintenance [7]. In cutaneous squamous cell carcinoma (cSCC), CD271 down-regulation may be involved in the transition from low to high-risk cSCC development [10]. On the other hand, its overexpression or activation strongly reduces cSCC aggressiveness in 3D and zebrafish models, thus becoming a significant target for this type of cancer [10].

To study the role of CD271 in pathological disorders of the nervous system [11], several CD271 knockout mouse models were developed [12–14]. However, none of them elucidate its precise functions in the skin. Herein, we have developed and characterized novel conditional constitutive or inducible epidermal-specific CD271-deleted mice, hereafter named cKO or icKO, respectively, where CD271 is absent in keratin 14 (KRT14) positive epidermal cells and their derivatives [15]. The most striking defect in the cKO mice was the altered skin homeostatic balance due to a major keratinocyte proliferation with a concomitant dysregulation of the differentiative process. We found a significant modulation of the molecular signaling associated with mitogenic pathways and an increased skin inflammation. Our models identify CD271-dependent signaling as critical for skin homeostasis, thus representing novel essential tools for studying skin pathophysiology linked to CD271.

## Results

### Epidermal deletion of CD271 leads to altered skin organization and dysregulation of epidermal proliferation and differentiation

**Fig. 1:**
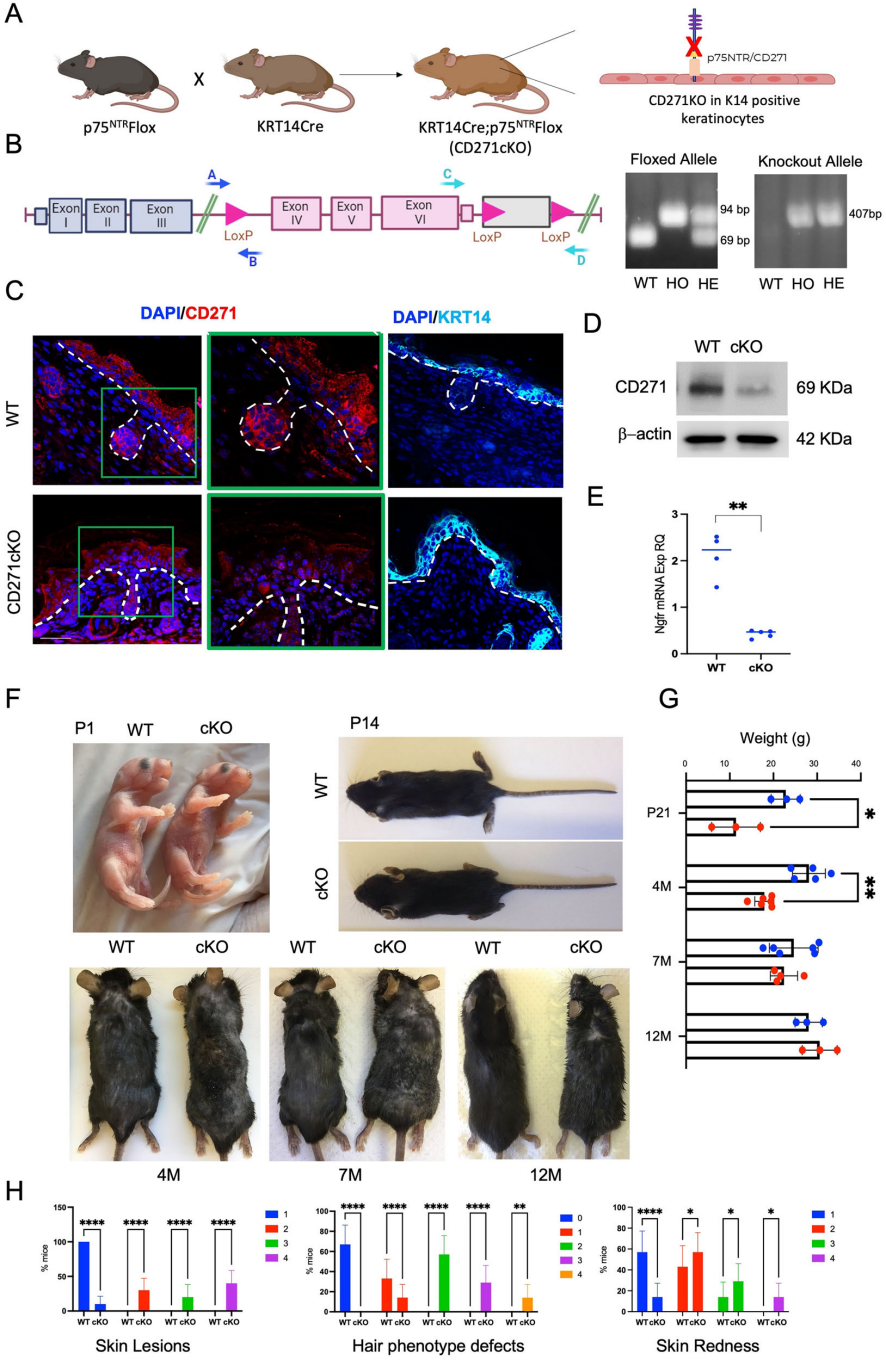
Epidermal deletion of CD271 in the skin leads to altered epidermal phenotypical characteristics during mouse development. **A** Schematic representation of mice mating and generation of cKO. **B** Scheme of the *Ngfr* gene and LoxP site (left panel) and mouse genotyping (right panel) (WT; wild type, HO; homozygous, HE; heterozygous). **C** Immunofluorescence of CD271 (red, right panel) and KRT14 (light blue, left panel) in cKO vs WT P1 (P=postnatal day) skin. Scale bar 50 μm. **D** Western blot analysis and **E** Real Time PCR of CD271 expression in cKO vs WT P1 skin. β-actin was used as reference protein and RPLPO as housekeeping gene. **F** Evaluation of the gross phenotype of cKO vs WT mice from P1 to 12 M (M=month) of age. **G** Evaluation of weight (g) of cKO vs WT mice of different ages. **H** Evaluation of skin lesions, hair phenotype defect, and skin redness in cKO vs WT 4 M mice. For skin lesions and hair phenotype defects, the score was given according to the % of body area involved: 0: no defects (for hair phenotype defects only) (blue); 1: 0–20% (red); 2: 20–40% (green); 3: 40–70% (violet), 4: 70–100% (orange). A score from 1 to 4 has been given for skin redness according to the severity of the redness and the % of the body area (1: 0–20% (blue); 2: 20–40% (red); 3: 40–70% (green), 4: 70–100% (violet). Data are presented as mean ± SD from three independent experiments. Statistical analysis was performed using the T-test analysis or two-way ANOVA. *: 0.01 < *P* < 0.05; **: 0.01 < *P* < 0.001; ****P < 0.0001.

CD271cKO (cKO) mice carrying a conditional deletion of CD271 in KRT14-expressing keratinocytes (Fig. 1A), were generated by mating constitutive KRT14-Cre with p75NTR^FX^ mice (Fig. 1B, left). CD271 KO was identified by genotyping (Fig. 1B, right panels) and CD271 deletion was validated at the protein and mRNA level in postnatal day 1 (P=postnatal day) skin (Fig. 1C–E). CD271 expression overlapping KRT14 expressing epidermal keratinocytes was also confirmed in the WT epidermis (Fig. 1C).

cKO mice are viable and fertile, generating a regular number of pups. Gross morphological analysis from P1 (P = postnatal day) to 12 months (M=months), showed no detectable differences between cKO and WT animals at P1 (Fig. 1F); however, a progressive development of hyperplastic skin features starting at 4 M of age, when spontaneous skin lesions, including scaly skin with scars and small ulcerations, skin redness, and/or hair phenotype defects, were seen mostly on the cKO dorsal skin (Fig. 1F–H). Overall, cKO mice presented significantly lower body weight as compared to WT animals (Fig. 1G). Altogether, these results strongly indicate that CD271 KO provokes significant skin alterations.

### Loss of CD271 is associated with epidermal hyperplasia and hair follicle impairment

**Fig. 2:**
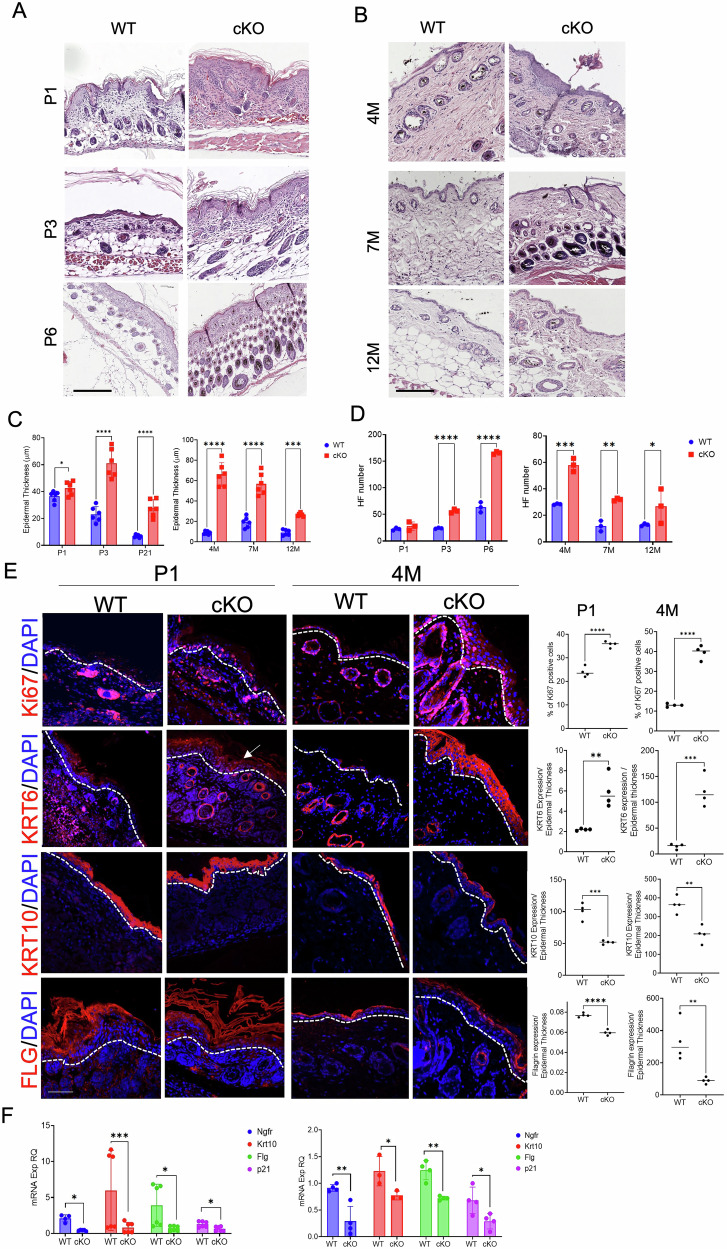
CD271 deletion induces skin disorganization and dysregulation of epidermal proliferation and differentiation during mice development. **A, B** Histological images (Hematoxylin and eosin – H&E -staining) of cKO vs WT skin of from P1 to 12 M of age. Scale bar 50 μm **C, D** Quantification of epidermal hyperplasia (thickness) and number of hair follicles in cKO vs WT skin from P1 to 12 M of age. **E** Representative images (Left panel) and relative quantification (Right panel) of the immunofluorescence staining for proliferation (Ki67 and KRT6) and differentiation (KRT1 and FLG) marker expression in cKO vs WT skin at P1 and 4 M of age (Left panel). Scale bar 50 μm **F** Evaluation of Ngfr, Krt10, Flg and p21 mRNA expression by Real-Time PCR. RPLPO as housekeeping gene. Data are presented as mean ± SD from three independent experiments. Statistical analysis was performed using the T-test analysis or two-way ANOVA *: 0.01 < *P* < 0.05; **: 0.01 < *P* < 0.001; ***0.001 < *P* < 0.0001; ****P < 0.0001.

Histological skin characterization was performed on P1, P3, and P6 skin, representing postnatal epidermal and hair development, and on 4 M, 7 M, and 12 M skin (Fig. 2A–D). From P1, cKO skin appears to be highly disorganized (Fig. 2A), with enlarged keratinocytes losing their typical palisade disposition (Fig. 2A, B, and Fig. S1). Epidermal thickness was significantly higher in cKO mice from P3 onward (Fig. 2C). Hair follicles (HFs) increased in number (Fig. 2D), with less uniform distribution and development within the hypodermis (Fig. 2A, B, Fig. S1). By P6, these differences were still evident. From 4 M, cKO epidermis became more hyperproliferative and irregular, with denser keratinization compared to WT. Differences in epidermal thickness and HF number remained statistically significant at 7 M and 12 M, highlighting CD271’s role in keratinocyte differentiation and proliferation.

To better investigate the epidermal changes due to CD271 deletion, we analyzed proliferation and differentiation markers in P1 and 4 M skin (Fig. 2E) and in all the previously indicated time points (Fig. S2a, b). An increase in Ki67-positive cells, and a decrease of KRT10 and Filaggrin (FLG) was found in cKO epidermis, confirming the altered homeostatic balance due to CD271 deletion (Fig. 2E; Fig. S2a, b). KRT6, typically absent in physiological condition, but expressed at elevated levels in psoriasis, wound repair, inflammatory states, and skin carcinoma [16], was expressed in cKO keratinocytes starting from P1, increasing up to 4 M (Fig. 2E, Fig. S2a-b), suggesting the development of an inflamed and activated skin phenotype [17, 18]. These data were confirmed at the transcriptional level (Fig. 2F); a reduced mRNA levels of Krt10, Flg and p21 (a p53-dependent negative regulator of the cell cycle [19]), was found in cKO skin, indicating impaired differentiation as compared to WT (Fig. 2F).

### Inducible CD271 deletion highlights murine skin alterations

**Fig. 3:**
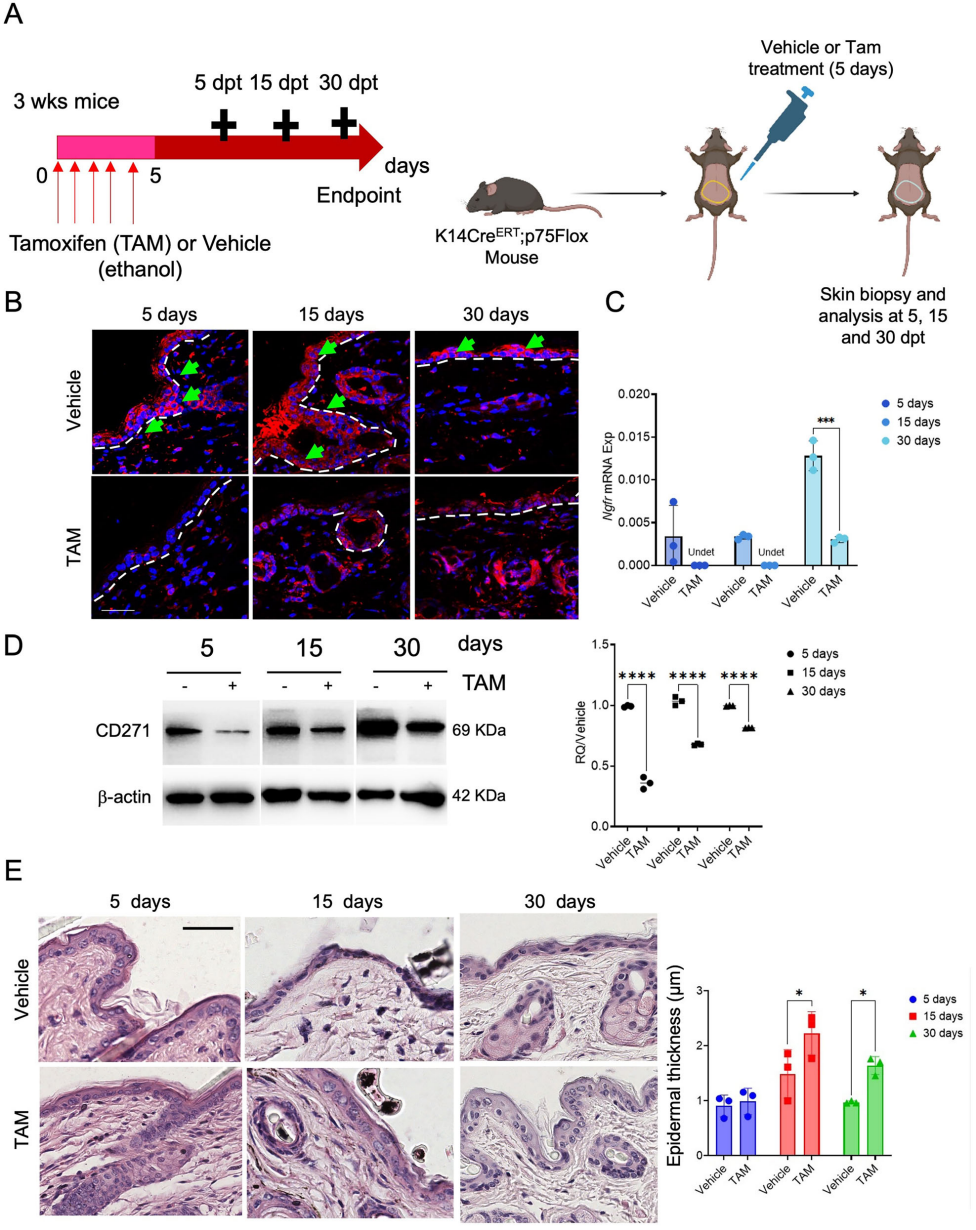
Inducible CD271 deletion provokes skin disorganization in adult mice. **A** Schematic representation of the tamoxifen or vehicle treatment protocol. (TAM = tamoxifen; wks = weeks, dpt = day post treatment) **B** Immunofluorescence analysis of CD271 expression (Red, green arrow) in TAM vs Vehicle treated animal at 5-, 15-, and 30-days dpt. Scale bar 50 μm. **C** Real Time PCR and **D** Western blot analysis of CD271 expression in TAM vs Vehicle treated animal at 5-, 15- and 30 dpt. β-actin was used as reference protein and RPLPO as reference gene. **E** Histological images (H&E staining) (left panel) and quantification of skin hyperplasia (skin thickness) (Right panel) in TAM vs vehicle-treated animal at 5, 15, and 30 dpt. Scale bar 50 μm. Data are presented as mean ± SD from three independent experiments. Statistical analysis was performed using two-way ANOVA. *: 0.01 < *P* < 0.05; ***0.001 < *P* < 0.0001; ****P < 0.0001.

The impact of CD271 deletion in adult skin was assessed using a novel CD271icKO (icKO) mouse developed with tamoxifen (TAM) responsive Cre expression in KRT14 positive cells. CD271 levels were significantly reduced at all time points, as indicated in Fig. 3A, and analyzed by IF, real-time PCR, and western blot (Fig. 3B–D; Fig. S3a). The icKO epidermis became progressively thicker, with major differences observed at 15 days, along with altered keratinocyte morphology (Fig. 3E, Fig. S3b). The icKO keratinocytes exhibited atypical features, including an altered nucleus-to-cytoplasm ratio and the presence of a peri-nuclear halo (Fig. 3E, Fig. S3b).

**Fig. 4:**
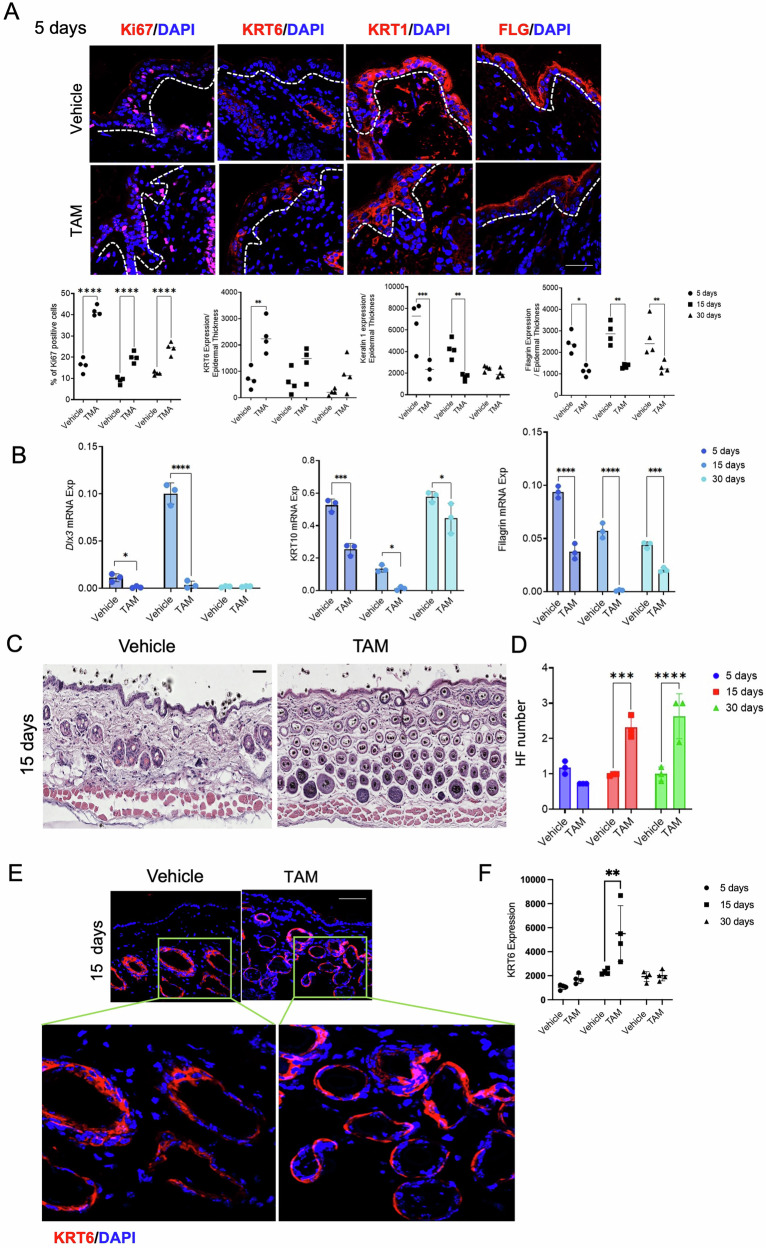
Inducible CD271 deletion causes dysregulation of skin proliferation and disorganization and an increased number of hair follicles in adult mice. **A** Representative image (Upper panel) and relative quantification (Below panel) of the immunofluorescence analysis of proliferation (Ki67 and KRT6) and differentiation (KRT1 and FLG) markers in TAM vs. vehicle-treated animal at 5 dpt. Scale bar 50 μm. **B** Evaluation of Dlx3, Krt10 and Flg mRNA expression by Real-Time PCR in TAM vs Vehicle treated animal at 5-, 15- and 30 dpt. RPLPO was used as reference gene. **C** H&E staining of TAM vs. vehicle-treated animal at 15 dpt. Scale bar 50 μm. **D** Quantification of number of hair follicles in TAM vs vehicle-treated animal at 15 dpt. **E** Representative images (left panel) and **F** fluorescence quantification (ImageJ software) of KRT6 expression in TAM vs vehicle animal hair follicles at 15 dpt by immunofluorescence. Scale bar 50 μm. Data are presented as mean ± SD from three independent experiments. Statistical analysis was performed using two-way ANOVA. *: 0.01 < *P* < 0.05; **: 0.01 < *P* < 0.001; ***0.001 < *P* < 0.0001; ****P < 0.0001.

icKO epidermis showed an increased Ki67-positive cells and reduced KRT1 and FLG (Fig. 4A and Fig. S4a, b). Keratinocytes also expressed KRT6 (Fig. 4A, Fig. S4a), indicating epidermal “activation” after CD271 deletion [16]. This pattern was confirmed at the mRNA level by the evaluation of Krt10 and Flg expression (Fig. 4B). The mRNA expression of DLX3, which is implicated in both the regulation of epidermal differentiation and HF development [19, 20], was strongly reduced at all time points (Fig. 4B).

Histology of transverse icKO skin sections showed an exacerbated development of HF structures (Fig. 4C), confirming our results in the cKO skin. At 15 and 30 days, where the effect of CD271 deletion is more evident, HF number was significantly higher in TAM-treated groups as compared to controls (Fig. 4C). Moreover, at 15-day post-treatment, KRT6 was strongly expressed in total icKO skin as compared to WT (Fig. 4E, F, Fig. S5a, b) highlighting HF altered structure, including immature morphology, and distribution compared to WT (Fig. 4E). This phenotype may potentially reflect a stage-dependent defect wherein CD271 loss disrupts the proper HF maturation. Further work will be needed to determine the exact CD271-dependent molecular signaling involved in HF differentiation.

### CD271 loss in keratinocytes modulates the activation of the mitogenic pathways and correlates with impaired skin barrier structure mimicking human skin diseases

**Fig. 5:**
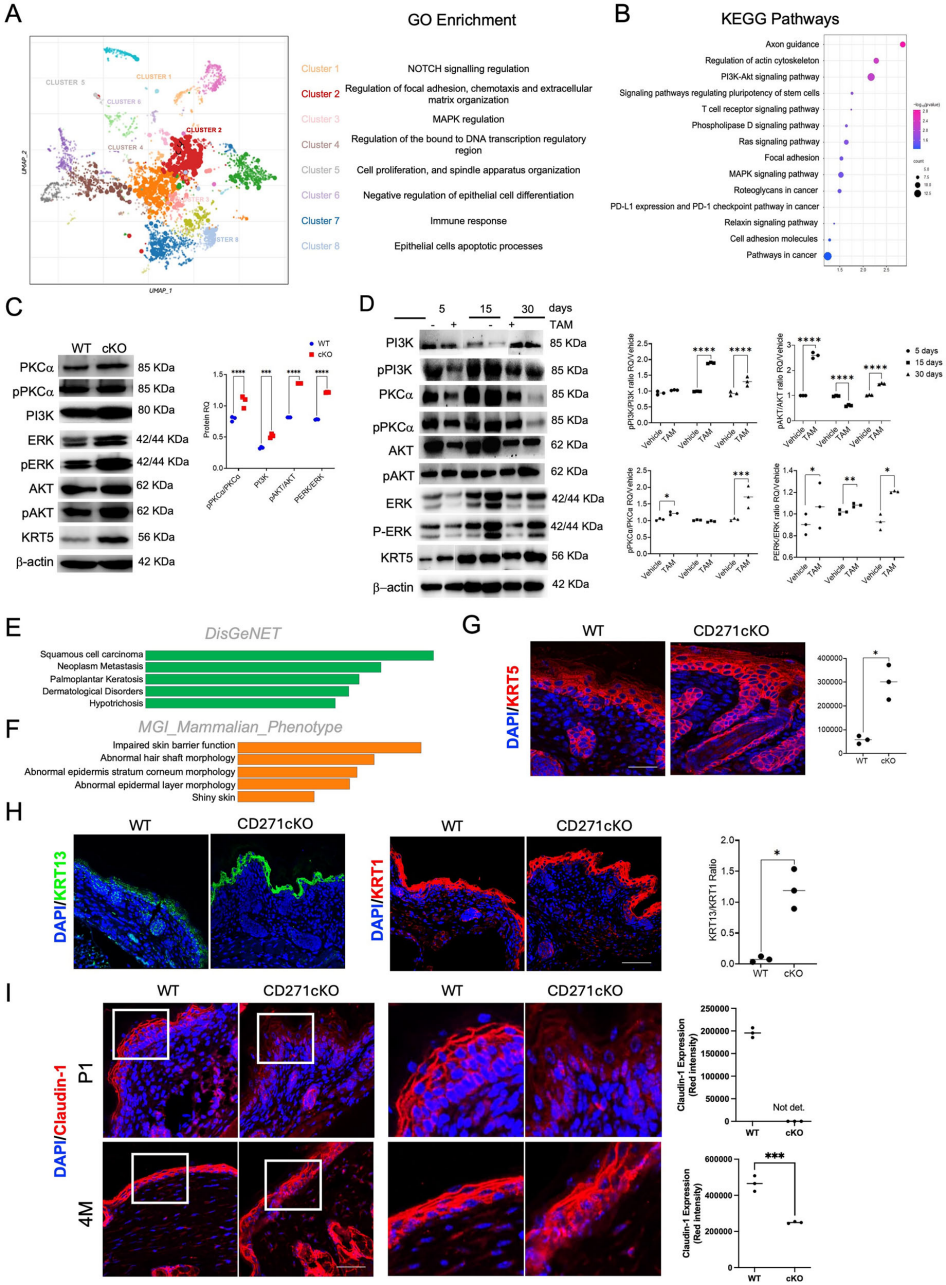
CD271 loss leads to a profound alteration of the transcriptomic profile and modulates the activation of the PI3K/Akt and ERK1/2 mitogenic pathways. **A** Scatter Plot of Total GO Term Enrichment. Each point represents a term in the gene set library. Term frequency-inverse document frequency (TF-IDF) values were computed for the gene set corresponding to each term, and UMAP was applied to the resulting values. Cluster 1: NOTCH signaling regulation; Cluster 2: Regulation of focal adhesion, chemotaxis, and extracellular matrix organization; Cluster 3: MAPK regulation, Cluster 4: Regulation of the bound to DNA transcription regulatory region. Cluster 5: cell proliferation, and spindle apparatus organization. Cluster 6: negative regulation of epithelial cell differentiation. Cluster 7: Immune response. Cluster 8: Epithelial cells apoptotic processes. **B** Enrichment pathways analysis through KEEG database of cKO vs WT significantly modulated mRNA. **C** Western blot analysis (left panel) of the activation of PI3K/Akt and PKCα/ERK mitogenic pathways in cKO vs WT total skin. On the right is reported the densitometric analysis and the ratio between the phosphorylated and not phosphorylated proteins. β-actin was used as reference protein. **D** Western blot analysis (left panel) of the activation of the mitogenic pathways in TAM vs Vehicle treated animals at 5, 15 and 30 dpt. On the right is reported the densitometric analysis and the ratio between the phosphorylated and not phosphorylated proteins. β-actin was used as reference protein. **E**, **F** Disease and phenotype profile enrichment performed by Enrichr against the DisGeNET and MGI_Mammalian_Phenotype libraries, respectively. **G** Representative images (left panel) and relative quantification (right panel) of KRT5 expression in WT vs cKO mouse skin at P1. Scale bar 50 μm **H** Representative immunofluorescence images of KRT13 (left panel, green) and KRT1 expression and quantification of the KRT13/KRT1 ratio (right panel) in cKO vs WT P1 skin. Scale bar 50 μm. **I** Representative immunofluorescence images of Claudin-1 (left panel) expression and quantification (right panel) in cKO vs WT P1 and 4 M skin. Scale bar 50 μm. Statistical analysis was performed using two-way ANOVA. *: 0.01 < *P* < 0.05; **: 0.01 < *P* < 0.001; ***0.001 < *P* < 0.0001; ****P < 0.0001.

To investigate the transcriptional programs that depend on CD271 expression, we performed transcriptomic profiling of cKO vs WT P1 skin (Fig. 5A, B; Fig. S6), which identified 444 differentially expressed genes (Fig. 5A, B; Fig. S6 and S7a). Single-cell clustering highlighted eight distinct Gene Ontology (GO) clusters, which revealed functional heterogeneity across cell populations (Fig. 5A). Moreover, by KEGG pathway analysis of differentially expressed genes (Fig. 5B), we significantly identified terms such as *Pathways in cancer*, *PI3K-Akt* and *MAPK signaling pathways* (Fig. 5B; Fig. S6 and S7a), known as crucial for cell proliferation and in several human skin diseases [21–23], including skin neoplasm [24]. Therefore, given their functional impact on skin, we validated PI3K and PKC pathway activation in our models, where an increase in PKCα and PI3K activation in cKO skin vs WT was found (Fig. 5C). Similarly, their main downstream effectors, ERK and AKT, respectively, showed a significant increase in expression and activation (Fig. 5C), confirming PI3K and MAPK pathway dysregulation in the cKO skin. Comparable results were obtained in icKO skin (Fig. 5D). The activation of the PI3K pathway increased in icKO skin at 15- and 30 days post-TAM, with a peak at 15 days. AKT activation was significantly increased at 5- and 30 days post-treatment, while no difference was observed at 15 days, suggesting additional positive or negative regulators of AKT at this time point. Most likely, the higher P-AKT level at 5 days post-treatment is due to the increased inflammatory condition after TAM treatment, at 15 and 30 days. Furthermore, given that this pathway is important for the barrier function of the stratum corneum, as well as for the maintenance of skin homeostasis [25], its alteration in icKO skin is compatible with the CD271-dependent epidermal abnormalities, as reported in Figs. 2–4.

Within the top KEGG terms (Fig. 5B), we also found the *Ras signaling pathway*, which plays a key role in epidermal homeostasis, and its constitutive activation leads to hyperproliferation with the development of skin lesions [26]. Furthermore, several genes are included in the *Axon guidance* pathway. In healthy skin, NGF stimulates the branching of sensory axons; in fact, NGF overexpression leads to increased innervation in murine skin [27]. Finally, *Regulation of actin cytoskeleton* was also identified in cKO vs WT skin transcriptome analysis. Given its role in the regulation of KSC [28], we could hypothesize that the CD271 expression regulates migratory signals, which could also be upregulated in invasive cells [29] (Fig. 5B and Fig. S6c, d and S7a). Moreover, we found a modulation of several genes implicated in the *NOTCH signaling pathway*, which is known to be important in the maintenance of human keratinocyte stemness [30], while its dysregulation is implicated in cutaneous cancer [31] (Fig. 5A, Fig. S6e). Additionally, several genes were involved in the *Epithelial Mesenchymal Transition pathway, TGFβ signaling* and *Inflammatory Response* (Fig. S6e and S7a). Upregulation of TGFb mRNA and several proliferation-associated genes, including MEK2 and Integrins (e.g. ITGA5, ITGB8), was corroborated by Tgif1 and PCNA mRNA levels (Fig. S7b). Similarly, we found the upregulation of Wnt5a, which plays a role in epidermal differentiation and stratification, and of Lpar4, which is associated with the WNT pathway and plays a role in skin appendage formation [32] (Fig. S7a). Our results strongly support the idea that CD271 modulates several targets in the context of an “active” - hyperproliferative and less differentiative skin, potentially correlating gene expression changes in the CD271cKO murine model to various human dermatological diseases.

To further address this point, we interrogated DisGeNet and MGI_Mammalian_Phenotype databases by using Enrichr (Fig. 5E, F). The enrichment analysis revealed direct links to human skin conditions such as cSCC, keratosis, and alopecia (Fig. 5E). These data were further supported by key marker analysis in murine skin. KRT5 expression was significantly increased in the cKO skin vs WT (Fig. 5G, Fig. S7c). While KRT5 is localized in suprabasal layers of the cKO skin, in WT mice, it is primarily confined to the basal layer (Fig. 5G). cKO also presented a higher expression of KRT13, correlated with keratinocyte proliferation, and a higher KRT13/KRT1 ratio, which is linked with malignant conversion in tumors [33] (Fig. 5H).

Moreover, we found gene terms associated with impaired skin barrier function, abnormal hair shaft, and epidermal morphology (Fig. 5F), confirming the existence of a strong correlation between the loss of CD271 and dysregulation of skin homeostasis. The identification of shared genes and pathways (like extracellular matrix organization and keratinization) further reinforces this correlation, suggesting that the CD271 deletion in keratinocytes at an early developmental stage significantly impacts skin homeostasis and predisposes to conditions mirroring human dermatological pathologies.

To confirm these findings, we also analyzed additional epidermal markers involved in skin barrier function and alteration. Claudin-1 (CLDN1), a critical component of tight junctions and skin barrier integrity, was markedly downregulated in cKO skin compared to WT (Fig. 5I). This downregulation is consistent with the observed impaired barrier function and is in line with previous reports showing that reduced CLDN1 expression leads to increased transepidermal water loss and skin inflammation [34]. In contrast, β-defensin, an antimicrobial peptide involved in innate immune defense, was significantly upregulated in cKO skin. The increased expression of β-defensin is indicative of an inflammatory microenvironment and has been associated with hyperproliferative and barrier-disrupted skin conditions such as psoriasis and atopic dermatitis [35] (Fig. S8a–b). Its induction likely reflects compensatory innate immune activation in response to compromised barrier integrity. Similarly, Toll-like receptor 2 (TLR2), a sensor of microbial components and a key regulator of innate immune responses in the skin, was also upregulated in the cKO. TLR2 overexpression has been reported in various skin disorders, including atopic dermatitis and psoriasis, where it contributes to inflammation and abnormal differentiation [36].

Finally, the cKO skin molecular data were also confirmed by the transcriptomic analysis of the adult skin derived from mice harboring the *Ngfr*^*tm1Jae*^ mutation [37], where, similarly to the cKO mouse model, KEGG analysis identified *Pathways in cancer*, *PI3K-Akt Signaling pathways*, and adhesion or inflammation-associated pathways (Fig. S9a). These data were further confirmed by Panther analysis (Fig. S9b). Specifically, a significant dysregulation of Lce, Collagen, and integrin-expressing genes was found (Fig. S9c).

Altogether, these findings reinforce the notion that CD271 deletion alters skin homeostasis through multiple converging mechanisms: impaired barrier function, aberrant differentiation, and activation of immune responses. These molecular alterations are coherent with the histological and phenotypic abnormalities observed in our models and reflect pathophysiological traits commonly reported in inflammatory and dysplastic skin conditions.

### CD271 null keratinocytes display increased viability by triggering mitogenic pathway activation

**Fig. 6:**
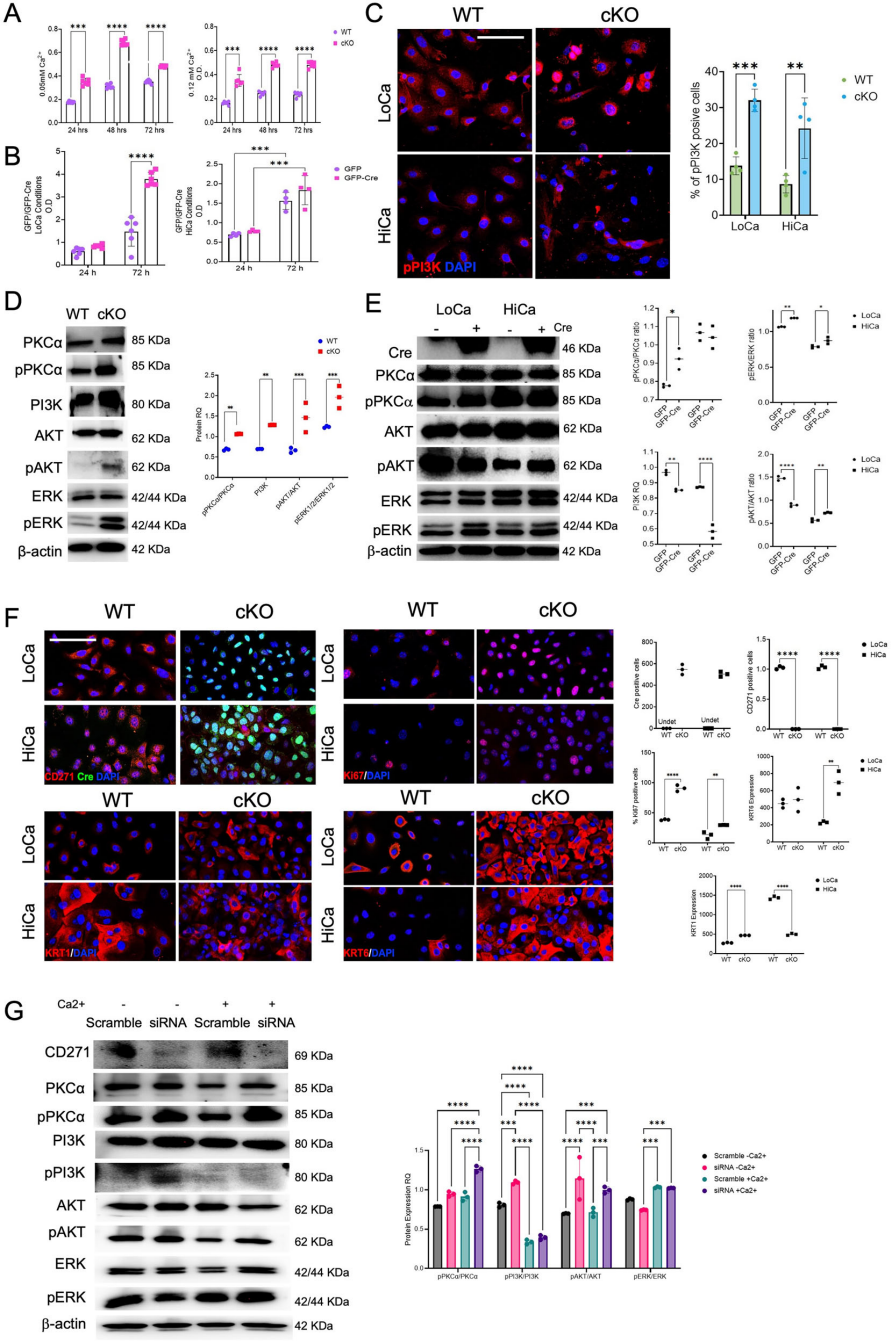
CD271 knock-out keratinocytes possess increased viability and resistance to differentiation stimuli by triggering mitogenic pathway activation. **A** MTT assay of cKO vs WT cells in Low Calcium (LoCa – 0.05 mM Ca^2+)^ and High Calcium (HiCa – 1.2 mM Ca^2+^) conditions. **B** MTT assay of primary mouse keratinocytes (PMK) infected with GFP or GFP-Cre viral vector in LoCa and HiCa conditions. **C** Immunofluorescence of phosphorylated PI3K (Red, Right panel) and relative fluorescence quantification (left panel) in cKO vs WT PMK. Scale bar 50 μm. **D** Evaluation of PI3K, PKCα, pPKCα, AKT, pAKT, ERK1/2 and pERK1/2 expression in cKO vs WT PMK by Western blot (Left). On the right were reported the relative densitometric analysis and the ratio between the phosphorylated and not phosphorylated proteins. β-actin was used as reference protein. **E** Evaluation of Cre, PI3K, PKCα, pPKCα, AKT, pAKT, ERK1/2 and pERK1/2 expression in PMK infected with GFP or GFP-Cre viral vector in LoCa and HiCa conditions by Western blot (Left). On the right were reported the relative densitometric analysis and the ratio between the phosphorylated and not phosphorylated proteins. β-actin was used as reference protein. **F** Evaluation of CD271, Ki67, KRT1 and KRT6 expression (Left panel) and relative quantification (Right panel) in cKO vs WT cells in LoCa and HiCa conditions. Scale bar 50 μm. **G** Evaluation of PI3K, PKCα, pPKCα, AKT, pAKT, ERK1/2 and pERK1/2 expression in mock vs CD271 silenced human keratinocytes in LoCa and HiCa condition by Western blot (Left). On the right were reported the relative densitometric analysis and the ratio between the phosphorylated and not phosphorylated proteins. β-actin was used as reference protein. Data are presented as mean ± SD from three independent experiments. Statistical analysis was performed using two-way ANOVA. *: 0.01 < *P* < 0.05; **: 0.01 < *P* < 0.001; ***0.001 < *P* < 0.0001; ****P < 0.0001.

To deeply dissect the characteristics of CD271 deleted keratinocytes, we analyze primary mouse keratinocytes (PMK), isolated as previously described [38]. After confirming CD271/Cre recombinase expression (Fig. S10a), viability was tested in PMKs maintained in both proliferative (0.05 mM Ca2 + ; LoCa) or differentiative media (0.12 mM Ca2 + ; HiCa). cKO PMKs seem to be less affected by the morphological changes induced by calcium (Fig. S10b). Independent of the culture conditions, cKO cells proliferate at a higher rate than WT PMKs (Fig. 6A). This effect is also maintained by the in vitro KO of CD271 using adenoviral vectors expressing Cre recombinase (Fig. 6B), showing that CD271 downregulation correlates with increased viability. GFP expression confirmed cell transduction (Fig. S10c).

In skin, the PI3K/Akt and PKCα signaling pathway is known to be involved in several processes, including proliferation, migration, differentiation, and metabolism [24, 39]. Upon CD271 deletion, cKO PMKs showed major PI3K phosphorylation, displayed as % of P-PI3K positive cells, as compared to WT, independently of calcium concentration in the medium (Fig. 6C). Similarly, PKCα level was upregulated in cKO cells as compared to WT (Fig. 6D). This data was also confirmed in Cre-deleted PMKs by western blotting, which shows higher expression of PKCα, ERK, PI3K, AKT and their phosphorylated isoforms (Fig. 6E) as compared to controls. In particular, CD271 depletion increases P-AKT in HiCa media, while LoCa Cre-positive cells display a lower phosphorylation state (Fig. 6E).

Under proliferative conditions, Ki67 expression was significantly higher in cKO PMKs than in WT. Following differentiation stimulus, Ki67 levels decreased in both groups but remained higher in cKO cells (Fig. 6F). Similarly, KRT6 was strongly expressed in cKO cells, confirming the in vivo results. Calcium addition reduced KRT6 expression in WT cells [16] but not in cKO cells, where KRT6 remained elevated regardless of LoCa or HiCa conditions (Fig. 6F). Conversely, the switch into an HiCa medium leads to an increase in KRT1 expression in WT cells, but not in cKO cells, where it showed no difference when compared to LoCa conditions. (Fig. 6F). These results show that CD271-deleted cells were less responsive to calcium-induced differentiation; exhibiting an activated profile confirmed by elevated expression of Ki67 and KRT6 in a medium condition that would physiologically drive differentiation.

To corroborate the correlation between CD271 and the previously described pathways in human skin, we analyzed the effect of CD271 silencing in primary human keratinocytes, maintained in low and high calcium conditions (Fig. 6G). Indeed, CD271 downregulation induced the increase of both PKCα and PI3K phosphorylation, as well as an increase of AKT and ERK activation and expression (Fig. 6G). Therefore, CD271 displays overlapping functions between humans and mice, thus further validating our models and their use as tools for studying skin diseases where CD271 and the neurotrophin network are involved [4].

### Mice lacking CD271 developed skin inflammation

**Fig. 7:**
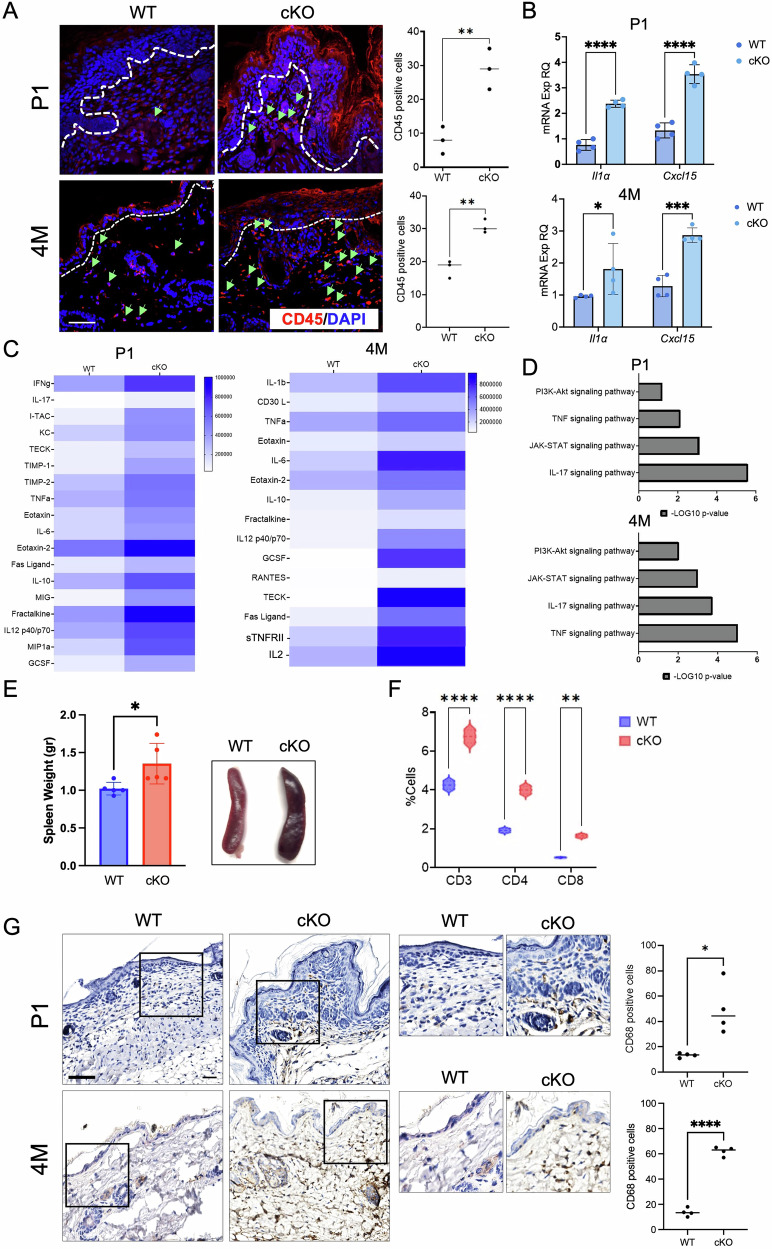
CD271 knockout determines the development of skin inflammation. **A** Evaluation of CD45 positive cells in cKO vs WT P1 and 4 M total skin (left panel) and relative quantification (right panel). Scale bar 50 μm. **B** Evaluation of IL1α and Cxcl15 (murine homolog of human IL8) mRNA expression in cKO vs WT P1 and 4 M total skin by Real-Time PCR. RPLPO was used as reference gene. **C** Heatmap representing the modulation of cytokine expression determined by Mouse Cytokine array in cKO vs WT total skin at P1 and 4 M of age and **D** relative pathways enrichment by GO analysis. **E** Representative images (Right) and relative weight (Left) of cKO vs WT mouse spleen at 4 M of age. **F** Cytofluorimetric analysis of CD3, CD4, and CD8 in cKO vs WT total skin at 4 M of age. **G** Representative images of CD68 expression (left panel) and quantification (right panel) in cKO vs WT P1 and 4 M skin by immunohistochemistry. Data are presented as mean ± SD from three independent experiments. Statistical analysis was performed using two-way ANOVA. *: 0.01 < *P* < 0.05; **: 0.01 < *P* < 0.001; ****P < 0.0001.

Similarly to other genetically engineered or chemically induced models resembling hyperproliferative skin conditions [40], the histological analysis of the cKO skin vs WT showed the presence of greater cellularity in the cKO dermis (Fig. 2A, B), potentially due to major inflammatory cell recruitment. Therefore, we investigate the expression of immune cell markers, such as CD45, expressed by nucleated hematopoietic cells [41]. A higher number of CD45-positive cells was detected in cKO skin as compared to WT, in both conditional (Fig. 7A) and inducible model (Fig. S11a, b). Interestingly, the number of CD45-positive cells in TAM vs vehicle-treated murine skin significantly increase at 15 and 30 days (Fig. S11a, b), suggesting that this not a primary event as compared to the dysregulation of differentiation markers (Fig. 4A, B).

Consistent with this finding, we detected an increased expression of Il1α and Cxcl15 (murine homolog of IL8) mRNA in both P1 and 4 M cKO skin, supporting the development of inflamed skin with an “active” keratinocyte response (Fig. 7B). The association between IL1α and IL8 with tumor microenvironment and psoriasis has been extensively reported in human skin [42–44]. Moreover, IL8 has a role in activating endothelial cells in tumors and inducing the recruitment of immune cells to the malignant microenvironment. Similarly, IL1α, which is also highly expressed by keratinocytes, is crucial for cancer-associated inflammasome [45].

Indeed, utilizing a mouse inflammatory cytokine array in cKO and WT tissue samples, we demonstrated a release of several cytokines and chemokines (Fig. 7C), such as TNFα, IFNγ, IL-17, KC (murine homolog for CXCL1), CCL3 (MIP1a), CCL9 (MIG), IL6, IL10 and IL12 that are produced at increased levels in cKO skin, both at P1 and 4 M. While the role of IL6 and IL12 in psoriasis is well-known [46, 47], MIP1a (CCL3) has shown a role in neoplasia and autoimmune skin diseases [48, 49]. MIG (CXCL9) is also associated with cellular infiltration in tumors [50], and Eotaxin is associated with tumor progression and Th2 diseases [51]. These results are compatible with both skin cancer and psoriatic phenotype [52, 53]. The analysis of the cytokine/chemokine profile indicated a correlation with the activation of the major pathways connected with epidermal hyperplasia, such as TNFα, IL17, JAK/STAT, and PI3K/Akt pathways [24, 54] (Fig. 7D).

We also observed major recruitment of inflammatory cells, including neutrophils, with the presence of necrotic collections within 4 M cKO lesions (Fig. S11c-d), macrophages, and lymphocytes (Fig. 7E–G and Fig. S11e-f). In detail, a splenomegaly, which indicates systemic inflammation, was found in cKO mouse as compared to WT (Fig. 7E). Moreover, we found a higher % of CD3, CD4, and CD8 positive cells in cKO vs WT mouse skin by cytofluorimetry (Fig. 7F) and an increased number of CD68 and CD163, common markers of macrophage, expressing cells in cKO skin as compared to WT (Fig. 7G and Fig S11e, f).

Taken together, our data indicate that the absence of CD271 expression in keratinocytes is associated with dysregulation of epidermal architecture and homeostasis, which might lead to the activation of multiple inflammatory pathways. As a result, cKO skin exhibits a pro-inflammatory environment that facilitates infiltration by different inflammatory cell types, a process observed in several pathological skin conditions.

## Discussion

Since their initial identification, an increasing body of evidence correlates the expression of NTs and their receptor to multiple functions in the human body [2, 3, 55]. Besides their crucial role in the development and function of the nervous system, NTs and their receptors are involved in a network at the skin level, where they act as key factors to favor skin homeostasis. Alterations of this network may lead to several pathological conditions [4]. CD271, together with Trk receptors, mediates many NT activities. However, CD271 can work independently, interacting with different ligands, and mediating both survival and apoptotic pathways [56], and its activity depends on the tissue and/or pathological context [4]. In psoriasis, CD271 exogenous reintroduction in keratinocytes can revert the psoriatic phenotype [7]. Also, CD271 activation in high-risk cSCC cells might revert to a low-grade phenotype, becoming a potentially effective therapeutic target for this type of tumor, especially in case of advanced disease [10]. Moreover, its potential use as a therapeutic strategy in combination with biological targeting Trk receptors has been demonstrated [10]. However, so far, no current models that directly allow the study skin physiopathologic processes correlated to CD271 have been developed. To fulfill this purpose, in the present work we have generated two CD271 KO models, characterizing their skin and keratinocytes.

Histological analysis identified a peculiar phenotype due to the absence of CD271. Consistently with the effect of CD271 activation or silencing in HIKs [5], we found an increase in epidermal thickness with an alteration of the epidermal structure and abnormal keratinocyte differentiation, starting from the basal compartment. After birth, the gene expression profile of the cKO skin indicates a defect of the molecular mechanisms regulating keratinocyte proliferation, differentiation and proper epidermal barrier formation. The upregulation of the KRT13 expression strongly suggests how, in the absence of CD271, keratinocytes are not able to control the mitogenic signaling events, and KRT13 can be found only in hyperproliferative contexts, in association with a malignant feature [19, 33, 57]. Furthermore, the dysregulation of Filaggrin and Claudin expression exacerbates barrier defects, which, combined with altered proliferation and differentiation, can drive the onset of an inflammatory phenotype.

Our results highlight a dysregulation of the PI3K/Akt pathway, as well as an increased activation of ERK signaling, thus producing “activated” or “initiated” keratinocytes, which are more prone to acquire differentiation defects and produce skin inflammation [17, 18, 58, 59]. The same pathways were altered in the skin of the CD271ciKO model and further supported by the full CD271 KO model analysis, where a significant alteration of PI3K/Akt pathway-associated genes, skin barrier, and of the extracellular matrix compartment was found. Additionally, PI3K and PKCα signaling alteration in primary keratinocytes demonstrates that CD271 might orchestrate several regulatory networks associated with proliferation, differentiation, and inflammation, and its absence potentially exacerbates the skin phenotype in response to external stimuli. Previous works demonstrate that CD271 causes the accumulation of PI3K molecules at the T cell membrane, influencing their activities [60]. We hypothesize that CD271 could exert similar effects in keratinocytes. KO mice could be more susceptible to developing the same type of defect in keratinocytes underlying psoriasis [61], such as KRT6 upregulation, KRT10 downregulation [62], and dysregulation of p15 and p21 proteins [63]. In particular, under physiological conditions, p21 transcription is regulated by the DLX3-p53 complex that increases during epidermal differentiation [64], while a close CD271-DLX3 connection has been shown in keratinocytes [38]. CD271 KO keratinocytes exhibit reduced expression of DLX3, which aligns with a low differentiation state and significant activation of PKCα pathways, as previously described [39]. An increased number of undifferentiated hair follicles was also detected in both the cKO and icKO mice. In particular, the hair follicles in TAM-treated CD271icKO mice appear morphologically in anagen, but their increased number and altered structure (including irregular spacing, immature bulb morphology, and ectopic expression of KRT6) suggest a disruption of the normal hair cycle, likely due to an incomplete or aberrant progression through differentiation stages. This phenotype may reflect a stage-dependent defect wherein CD271 loss disrupts the proper exit from telogen or the transition to catagen.

CD271 expression dysregulation has been previously detected in human inflammatory skin diseases [4], suggesting that the lack of CD271-associated functions is potentially involved in their pathophysiology. The cKO model developed spontaneous skin lesions, with a higher number of inflammatory cells infiltrating the skin, strongly correlating CD271 with the maintenance of skin integrity through the resident immune cell activities.

In the icKO model, CD45-positive cells increased in the icKO mouse skin at 15 and 30 days, but not at 5 days, indicating that inflammation occurs later, after CD271 knockout. In contrast, Filaggrin expression, which reflect of skin barrier integrity, dropped significantly as early as 5 days post-CD271 deletion. These results indicate that CD271 knockout in keratinocytes first causes epidermal barrier defects, which are then followed by skin inflammation. Substantially, these findings support a model in which loss of CD271 in keratinocytes provokes a primary epidermal defect, followed by local production of chemotactic factors that promote immune cell homing to the skin. Consistently, a marked upregulation of cytokine and chemokine was detected in the skin of cKO mice. However, follow-up studies employing inflammation-specific protocols and a larger cohort of animals will be required to fully elucidate the inflammatory landscape of CD271 KO skin.

By detailing CD271-dependent signals, our results provide novel insights for other conditions involving hair follicle dysregulation or skin infections [65]. Furthermore, the same pathways associated with CD271 knockout are also present in human keratinocytes, as CD271-silenced keratinocytes exhibited a similar expression profile to the murine KO keratinocytes, regardless of the calcium concentration in the media, allowing for translation from mice to humans. Simultaneously, the disruption of these signals leads to an inflammatory cell response both locally, at the skin level, and systemically, as indicated by splenomegaly. Based on these findings, CD271 appears to play a role in regulating epidermal homeostasis and barrier functions; its absence is associated with disruptions in epidermal regulation, which may contribute to the development of various skin disorders. Given the established role of CD271 in cSCC [10] and its clear molecular association with this pathology in the cKO model, we will intend to dissect its contribution to shaping a pro-tumorigenic milieu in a larger cohort of animals.

**Fig. 8:**
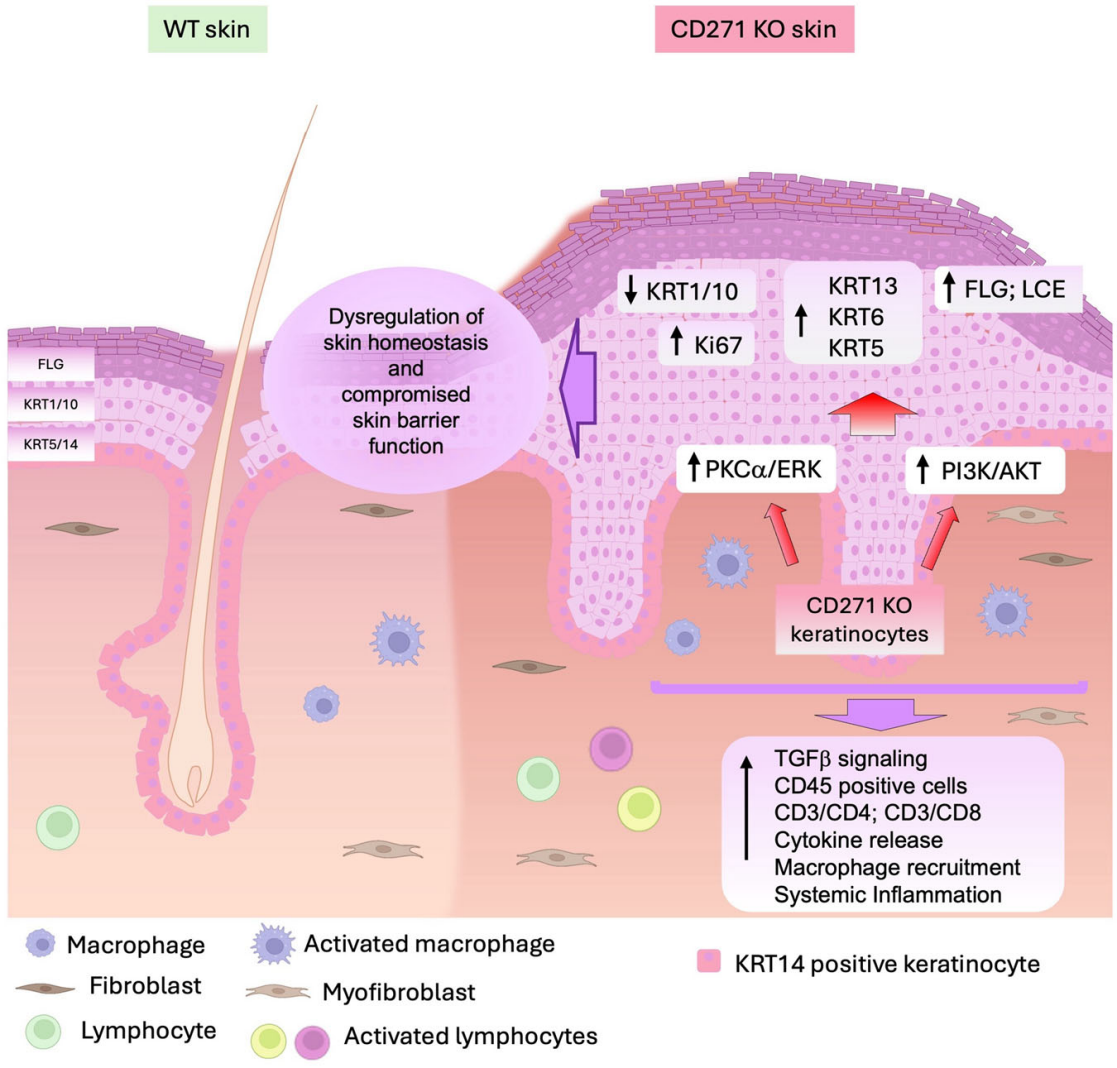
CD271 deletion in the skin triggers PI3K/Akt and PKCα/ERK pathways activities determining disorganization, altered differentiation, and inflammation. Normal mouse skin (WT) is divided into 3 main layers: the epidermis, the dermis, and the hypodermis. The epidermis consists of only 2–3 layers of cells. KRT14 is expressed in the basal layer, and, during the differentiation process, keratinocytes start to express KRT1/10 and, in the last layers, FLG. The dermis consists mainly of fibroblasts and the presence of a few inactive immune cells (principally, leukocytes and macrophages). On the other hand, CD271 KO skin is characterized by profound skin disorganization, and proliferation stimuli prevail. The absence of CD271 in keratinocytes provokes a hyperactivation of the PI3K/Akt and PKCα/ERK1/2 pathways provoking the boost of keratinocyte proliferation ability, by increasing the expression of Ki67, KRT6, KRT13, and KRT5, which in turn leads to an alteration of the differentiation process (decrease expression of KRT1/10, FLG, and resistance to differentiation stimuli). Altogether, these phenomena induce intense skin inflammation, with an increased expression of pro-inflammatory cytokine, the recruiting, and the activation of leukocytes and lymphocytes. Moreover, CD271 KO mouse skin shows an increased dermal density, mainly caused by the presence of myofibroblast increased production of extracellular matrix components (i.e. Collagen), and increased production of immature hair follicles.

In conclusion, these results strongly emphasize the role of CD271 in the skin, providing the first evidence that its absence leads to a significant disruption of skin homeostasis (schematically represented in Fig. 8). CD271cKO and CD271icKO mice are essential for unraveling the molecular pathways involved in skin maintenance and represent a valuable tool for studying CD271-mediated skin diseases, where the activation of CD271 pathways may offer a promising therapeutic strategy for skin conditions related to keratinocyte hyperproliferation and inflammation.

## Methods

### Study approvals

The animal studies described here were reviewed and approved (Aut. N. 405/2015-PR; 496-2020-PR; 737/2024-PR) by the Italian Ministry of Health according to the 3 R (replacement, reduction, and refinement) principles and guidelines established by the EU legislation on animals in science.

For the human cell study, informed written consent to the protocol was obtained for all subjects and was approved by the Ethical Committee of Area Vasta Emilia Nord (Prot. N. 353/2017).

### Mouse strain development and genotyping

C57BL/6 J mouse strain p75NTR^Flox^ (floxed homozygote) was kindly gifted by Dr Brian Pierchala and Dr Vesa Kaartinen, University of Michigan. These mice possess *loxP* sites flanking exons 4 through 6 of the targeted *Ngfr* gene [66]. C57BL/6 J K14-Cre strain, expressing Cre recombinase under the control of KRT14 promoter, was kindly gifted by Dr Maria I. Morasso, NIAMS/NIH. The K14-CreERT2 (Stock No: 005107; K14–CreERtam) was purchased by JAX Laboratories for research purposes. Mice were used to generate a K14Cre; p75NTR^Flox^ (CD271cKO) or K14CreERT2; p75NTR^Flox^ (CD271icKO) mouse strain, respectively. Genotype was assessed by PCR and oligos are reported in Table S1.

### Tamoxifen treatment

CD271ciKO dorsal skin was shaved 2 days before the application of 4-hydroxy-tamoxifen (4-OH tamoxifen, Sigma-Aldrich Co., St. Louis, MO). 1 mg/ml of 4-OH tamoxifen dissolved in ethanol 200 μl was topically applied to 3-week-old mice dorsal skin for five consecutive days. Skin samples were collected at 5-, 15-, and 30-day post-treatment.

### Primary mouse keratinocyte culture and transduction

Primary murine keratinocytes (PMK) were isolated from CD271cKO or p75NTR^Flox^ mice as previously described [19]. For each experimental replicate, a minimum of 6 newborn mice from independent breedings were used. PMK were cultured in S-MEM (Gibco, Thermo Fisher Scientific, Waltham, MA, USA) with 8% of chelexed plus 1% not chelexed Fetal Bovine Serum (FBS, Corning, New York, USA), 1% antibiotic (PSA, Sigma-Aldrich Co., St. Louis, MO), with Ca^2+^ concentration of 0.05 mM (referred to as LoCa medium). To induce keratinocyte differentiation, a final concentration of 0.12 mM Ca^2+^ was used (referred to as HiCa medium).

p75NTR^Flox^ PMK were transduced with GFP or Cre adenoviruses (50 MOI; Vector Biolabs, Malver, PA, USA), according to the manufacturer’s indication, in the presence of 4 μg/ml polybrene (Sigma-Aldrich Co., St. Louis, MO). GFP expression and brightfield images were taken by ZOE Biorad microscope.

### Human keratinocyte culture and transfection

Healthy Human Keratinocytes (HHK) were isolated from healthy skin biopsies and the isolated cells were cultured on 3T3 feeder layer, as previously indicated [67]. Passage 1 or 2 keratinocytes were plated and maintained in Keratinocyte Growth Medium (KGM) (Lonza, Clonetics, San Diego, CA, USA) and subjected to CD271 or scramble siRNA (Dharmacon Inc, Lafayette, CO, USA). 72 h after transfection, cells were collected for the subsequent experiment.

### Mouse skin histology, immunofluorescence and immunohistochemistry staining

Skin biopsies were obtained from mice at different ages as per experimental conditions. Skin sections were embedded in paraffin and 4 μm thickness sections underwent H&E staining or immunofluorescence as previously described [57]. More details are reported in supplementary materials. Primary antibodies were reported in Table S2. Pictures were taken by Leica Confocal Sp8 Microscopy. The Opti-View DAB immunohistochemistry automated detection kit (Ventana Medical Systems) was employed to CD68 and CD163 expression by the Department of Pathology of the University Hospital (Modena, Italy).

### Immunocytochemistry

PMK were isolated from WT or cKO murine skin at P1-P3 from three independent breeding. Following 24 h from the plating into 8-well chamber slides, the media was switched into LoCa or HiCa condition. After 48 h, cells were fixed in 4% paraformaldehyde, then a blocking solution of BSA 1% was added for 20’ and subsequently permeabilized with Triton X-100 for 5’. After washing, the cells were incubated with primary antibodies (Table S2) followed by staining with Goat anti-Rabbit IgG Secondary Antibody, Alexa Fluor 546 or 488 conjugate (Invitrogen, Carlsbad, CA, USA, dilution 1:500 in PBS). DAPI solution was added to each well and incubated for 2–5 min at room temperature. Images were taken with a Leica Sp8 Confocal microscope. Imaging analysis of at least 3 independent fields for 3 images was performed by ImageJ software.

### RNA extraction and qRT-PCR

Total RNA was extracted using RNeasy kit (Qiagen, Hilden, Germany) according to the manufacturer’s instructions. cDNA was prepared using the High-capacity cDNA RT kit (Applied Biosystem, Thermo Fisher Scientific, Waltham, MA, USA). For each gene, qRT-PCR analysis was done in triplicate using the DyNAmo Flash SYBR Green qPCR (Thermo Fisher Scientific, Waltham, MA, USA). Relative gene expression was determined based on the 2 − ΔΔCT method with normalization of the raw data to the murine RPLPO mRNA expression. Primer sequences are reported in Table S3.

### Transcriptome analysis

Five different mice per genotype were used for RNA-seq analysis from independent breedings. RNAseq was performed by Ion Torrent™ NGS technology with the Ion AmpliSeq Transcriptome panel Mouse Gene Expression CORE on GeneStudio S5™System platform (Thermo Fisher Scientific, Waltham, MA, USA). For the enrichment analysis of all the modulated mRNA, we used multiple tools, including David GO Functional analysis (https://davidbioinformatics.nih.gov), PANTHER GO (https://pantherdb.org/), and EnrichR, and SRplot (bioinformatics.com) was used to generate bubble charts. Disease and phenotype enrichment was performed using EnrichR against the DisGeNET and MGI_Mammalian_Phenotype libraries (https://maayanlab.cloud/Enrichr/). More information is reported in the supplementary materials.

### Western blotting

Total proteins were extracted with RIPA (radioimmunoprecipitation assay) lysis buffer containing protease inhibitors. Equal amounts of protein for each sample were run on a 6%–15% SDS–PAGE gel and transferred onto a nitrocellulose membrane. Membranes were incubated overnight at 4 °C with the primary antibodies, which are reported in Table S2. More details are available in supplementary materials.

### MTT assay

PMKs isolated from p75NTRFlox mice were seeded in two different media: HiCa and LoCa. Under both conditions, they were separately infected with two viral vectors, control adeno-GFP and adeno-Cre (Vector Biolabs, Malver, PA, USA). PMKs were transduced with vectors vehiculated from polybrene 8 μg/ml, at MOI of 50, 24 h after seeding post isolation in LoCa medium. The MTT assay was subsequently performed at 24 h post-infection to highlight changes in viability or cytotoxicity following deletion of the CD271 receptor, as previously indicated in supplementary materials.

### Cytokine array, lymphocyte isolation and FACS analysis

Mouse Inflammation Antibody Array-Membrane kit (Abcam, Cambridge, UK) was used on protein lysates obtained from P1 and 4 M cKO and WT skin, following manufacturer protocol. More details are reported in supplementary materials.

Lymphocyte isolation from cKO and WT skin biopsies were performed as previously described [54]. More details were indicated in the supplementary information.

### Statistical analysis

The number of animals for each experiment was determined according to the project Aut. N. 405/2015-PR; 496-2020-PR and 737/2024-PR, based on the guidelines established by the EU legislation on animals in science, reviewed and approved by the Italian Ministry of Health. More details are provided in the Supplementary Materials. Values in the figures are expressed as mean ± SD. Analysis was performed using GraphPad Prism 6.0. The significance of experimental differences was evaluated by an unpaired (two-tailed) Student’s *t* test, when comparing two groups. A one-way or a two-way ANOVA followed by a Tukey analysis was used when more than two groups were compared. Statistical significance is indicated by * symbols (*: 0.01 < *P* < 0.05; **: 0.01 < *P* < 0.001; ***0.001 < *P* < 0.0001; ****P < 0.0001) ns indicates not significant.

## Supplementary information

Supplementary Information and Supplementary Figures

Supplementary Materials

## Data Availability

Data supporting the findings of this study are available in the article, its Supplementary information, the source data file, and from the corresponding author upon reasonable request. RNA-sequencing data have been deposited in the Gene Expression Omnibus (GEO) database under accession code GSE288424.
