# Supplementary material for: CD271 orchestrates skin structure, differentiation, and inflammation via PI3K/Akt and PKCα/ERK pathways
Source: Cell Death Dis. 2025 Oct 21;16(1):735. doi: 10.1038/s41419-025-08062-5 (PMC12540997; doi:10.1038/s41419-025-08062-5)
Supplement: Supplementary file 1 — Supplementary Information and Supplementary Figures [file 41419_2025_8062_MOESM1_ESM.docx]

**SUPPLEMENTARY MATERIALS**

**METHODS**

**Mouse skin histology and immunofluorescence staining**

Skin biopsies were obtained from mice at different ages as per experimental conditions. Skin sections were embedded in paraffin, and 4 μm thickness sections underwent H&E staining or immunofluorescence as previously described (Bajpai et al., Oncogene). Following deparaffinization and rehydration, antigen unmasking with citrate buffer 1X (Bio-Optica) or EDTA buffer for 20 minutes at 98°C was performed. Then, slides were incubated with a blocking solution composed of 3% dry milk and 3% goat serum, in PBS, for 1 hour at room temperature. After blocking, the slices were incubated with the primary overnight at 4°C in a humidified chamber. Primary antibodies were reported in Table S2. Then, slices were washed three times with PBS-Tween (Tween 0.05%) buffer and incubated with the secondary antibodies (Alexa Flour 568 Goat-Anti-Rabbit secondary antibody, Invitrogen, diluted 1:500 in PBS) for 45 minutes at room temperature. After this step, treatment with DAPI (4′,6-diamidino-2-phenylindole,1 µg/µl in methanol-diluted 1:2000 in MilliQ-water) for 5 minutes was performed. Pictures were taken by Leica Confocal Sp8 Microscopy.

**Primary mouse keratinocyte culture and transduction**

Primary murine keratinocytes (PMK) were isolated from CD271cKO or p75NTR^Flox^ mice as previously described^20^. For each experimental replicate, a minimum of 6 newborn mice from independent breedings were used. PMK were cultured in S-MEM (Gibco) with 8% of chelexed plus 1% not chelexed Fetal Bovine Serum (FBS, Corning), 1% antibiotic (PSA, Sigma), with Ca^2+^ concentration of 0.05 mM (referred to as LoCa medium). To induce keratinocyte differentiation, a final concentration of 0.12 mM Ca^2+^ was used (referred to as HiCa medium).

p75NTR^Flox^ PMK were transduced with GFP or Cre adenoviruses (50 MOI; Vector Biolab), according to the manufacturer’s indication, in the presence of 4 μg/ml polybrene (Sigma). GFP expression and brightfield images were taken by ZOE Biorad microscrope.

**Transcriptome analysis**

Five different mice per genotype were used for RNA-seq analysis from independent breeding’s. RNAseq was performed by Ion Torrent™ NGS technology with the Ion AmpliSeq Transcriptome panel Mouse Gene Expression CORE on GeneStudio S5™System platform (Thermo Fisher Scientific). Total RNA was extracted using the Quick-DNA/RNA MiniPrep Plus Kit (Zymo Research). Samples were sectioned into three parts and added to the prefilled tubes of the Precellys Lysing Kit, adding 900 μL of DNA/RNA shield diluted 1:1 in water, 45 μL of protease inhibitor, and 90 μL of digestion buffer, and placed in the Precellys 24 rotor. The quality and quantity of extracted RNA were measured by calculating the fluorescence with Qubit (Thermofisher). RNA was reverse transcribed with Super Script VILO cDNA Synthesis Kit (Invitrogen). The cDNA was amplified by using the Ion AmpliseqTM 517 Transcriptome Mouse Gene Expression kit for library preparation, according to the manufacturer. The library was quantified by using the Ion Library TaqMan Quantitation kit (Thermofisher) in Real-Time PCR. For each sample, 70 pM were pooled for templating on One Touch 2 (OT2). After emulsion PCR and subsequent Enrichment, the Ion Sphere enriched was loaded onto Chip 540 for the sequencing. The reads have been aligned with the Mouse genome (Ensembl GRCh38) using Torrent Suite™ Software 5.8.1. The Coverage Analysis plugin has been used to create amplicon counts that have been imported into the TAC (Transcriptome Analysis Console) platform. Differentially expressed genes have been determined from adjusted p-values (<0.05) and Fold change (≥ 2), and hierarchical clustering was determined.

For the enrichment analysis of all the modulated mRNA, we used multiple tools, including David GO Functional analysis (<https://davidbioinformatics.nih.gov>), PANTHER GO (<https://pantherdb.org/>), and EnrichR, and SRplot (bioinformatics.com) was used to generate bubble charts. In detail, EnrichR^68^ consulting the following database: GO Biological_Process_2023, WikiPathways 2024 Mouse, KEGG 2021 Human, MSigDB Hallmark 2020. Differentially expressed genes were separated into downregulated (FoldChange>=2) and upregulated (FoldChange>=2) and subsequently for the enrichment analysis of mRNAs, we used the software STRINGdb^69^ (GO database). For the scatter plot, the Applyter tool was used after enrichment was processed on EnrichR, in which genes were listed according to similarity using the Leiden algorithm. Points are plotted on the first two UMAP dimensions. Disease and phenotype enrichment was performed using EnrichR against the DisGeNET and MGI_Mammalian_Phenotype libraries (<https://maayanlab.cloud/Enrichr/>).

**Western blotting**

Total proteins were extracted with RIPA (radioimmunoprecipitation assay) lysis buffer containing protease inhibitors. Equal amounts of protein for each sample were run on a 6%–15% SDS–PAGE gel and transferred onto a nitrocellulose membrane. Membranes were incubated overnight at 4 °C with the primary antibodies, which are reported in Table S2.

After 3 washes with a PBS/tween or TBS/tween solution, membranes were incubated with secondary antibodies, goat anti-Mouse or goat anti-Rabbit (1:3000; Bio-Rad Laboratories), for 45 min at room temperature. Bands were visualized with a chemiluminescence detection system (Amersham Biosciences UK Limited, Little Chalfont Buckinghamshire, UK). Each experiment was performed in triplicate with different experimental samples. Densitometry analysis of the bands was performed by ImageJ software and data are presented as mean ± SD.

**MTT Assay**

The MTT solution was prepared at a concentration of 1mg/1ml in S-MEM medium and 50 ul of the solution is added to each well and incubated at 37°C for two hours. Afterward, the medium is aspirated and 100 ul of isopropanol is added, to solubilize the salt. The resulting solution is read on a spectrophotometer (iMark™ Microplate reader, Bio-Rad) at a wavelength of 540 nm.

**Cytokine array**

Mouse Inflammation Antibody Array-Membrane kit (Abcam) was used, following manufacturer protocol. Briefly, cKO and WT skin were homogenized, and lysed by using the supplied 2X lysis buffer, previously diluted to a concentration of 1:1 with deionized water and supplemented with protease inhibitors. Following the protein extraction process, the samples were diluted to a final volume of 1 ml with 1X blocking buffer and 300 μg of total protein per membrane were used.

Each membrane was then blocked with 2 mL of 1X blocking buffer provided by the kit for 30 min. 1 mL of the previously prepared material was transferred into each well and incubated overnight at 4°C. The membranes were then incubated with 1 mL of 1 X anti-cytokine-conjugated biotin solution at room temperature for 2 hours and washed as before. Finally, 2 mL of HRP-conjugated streptavidin was introduced for 2 hours. Bands were visualized with a chemiluminescence detection system (Amersham Biosciences UK Limited, Little Chalfont Buckinghamshire, UK).

**Lymphocyte isolation and FACS analysis**

cKO and WT skin biopsies (about 2cm) were cut and incubated in a digestion mix composed of Collagenase I and DNasi I in RPMI basal medium at 37°C for 2 hours in gentle agitation, as previously described^52^. Subsequently, the cellular suspension was filtered with a 100µM cell strainer and blocked with RPMI with 5% of FBS and then filtered with a 70 and 40µM cell strainer and centrifuged at 1800 rpm for 8 minutes.

Cells underwent a pre-incubation step with the antibody anti-CD16/CD32 (Thermo Fisher Scientific; diluted 1:100) for 20 minutes at 4°C and, subsequently, were incubated with the primary antibody Mouse anti-CD3-PE, Mouse anti-CD4-PE Cyanine7 and Mouse anti-CD8-PE cyanine 5 (all (Thermo Fisher Scientific) diluted 1:100 in the eBioscience Flow Cytometry Staining Buffer (Thermo Fischer Scientific) for 30 minutes at 4°C. Cells were then centrifuged at 400g for 5 minutes at room temperature and resuspended in the staining buffer.

The compensation matrix was performed by using the UltraComp eBeads™ Plus Compensation Beads (Thermo Fisher Scientific), following the manufacturer instructions. Cytofluorimetric analyses were performed by using Attune™ Flow Cytometer (Thermo Fisher Scientific).

**Statistical analysis**

The number of animals for each experiment was determined according to the project Aut. N. 405/2015-PR; 496-2020-PR and 737/2024-PR, based on the guidelines reviewed and approved by the Italian Ministry of Health. Briefly, for the evaluation of proliferation, differentiation, and immunological markers in skin biopsies, by the described techniques, sample size was chosen to ensure adequate power to detect a pre-specified effect size. In particular, Ki67 expression (cell proliferation index) was analyzed as the primary outcome by two‑way ANOVA considering the independent factors “Model” (2 groups; WT or cKO) and Time (3 groups; 1, 3 and 6 Postnatal days). Same consideration was made for 4, 7, and 12 months of age. Based on the data reported by Palazzo et al. (2017) in Cell Death & Differentiation, we assume that Ki67 counts within each group are approximately normally distributed with a standard deviation of 1. In the control group, the mean Ki67 value is assumed to be 10 cells per field, and we anticipate a minimum percentage change of 30% in at least one of the other groups (corresponding to a minimum Cohen’s f of approximately 0.47). With a two-sided type I error rate of 5% and 80% power, the required sample size was 36 animals. Therefore, three independent replicates with 6 animals (*n*=6) for each genotype were performed. Transcriptome analysis was made with 5 different mice per genotype from independent breeding. For TAM treatment, similar statistical considerations were made. No blinding was performed.

The planned number of animals considered the dropout rate, in case of behavior-dependent superficial skin abrasions, triggering physiological wound-healing responses and introducing additional experimental variability (e.g., elevated inflammatory mediators) that could affect the outcome measures.

Concerning in vitro experiments with PMK, the level of keratinocyte proliferation assessed by MTT assays at two different time points was considered. Based on the data reported by Zang et al. (2012) in Journal of Cell Science, we assume that the Optical Density (correlated with cell proliferation) within each group is approximately normally distributed with a standard deviation of 0.05, and that the control group mean is 1. With a two-sided type I error rate of 5% and 80% power, the required sample size is, for each experiment, 16 animals in total (i.e., 4 animals per group). For human keratinocytes, similar considerations were made; three independent replicates with keratinocytes isolated from healthy skin biopsies and processed as described in (67) were done.

Table S1**:** Primers sequence for mice genotyping

| Cre-F | TCT GGC ATT TCT GGG GAT TGC |
| --- | --- |
| Cre-R | CTA ATC GCC ATC TTC CAG CAG |
| CreERT-F | CGC ATC CCT TTC CAA TTT AC |
| CreERT-R | GGG TCC ATG GTG ATA CAA GG |
| P75Flox-F | TGC AGA AAT CAT CGA CCC TTC CC |
| P75Flox-R | TCC TCA CCC CGT TCT TTC CCC |
| P75KO-F | CCT CCG CCA GCT GTC TGC TTC CT |
| P75KO-R | TCC TCA CCC CGT TCT TTC CCC |

Table S2**:** Primary antibodies

| **Antibody name** | **Use (dilution)** | **Manufacturer** |
| --- | --- | --- |
| Rabbit anti-Ki67 | Immunofluorescence and Immunocytochemistry (1:100) | Bethyl Laboratories IHC‑00375 |
| Rabbit anti-K6 | Immunofluorescence /Immunocytochemistry (1:100) and Western blot (1:1000) | BioLegend 905701 |
| Rabbit anti-K1 | Immunofluorescence and Immunocytochemistry (1:100) | BioLegend 905201 |
| Rabbit anti-Fillagrin | Immunofluorescence (1:100) | BioLegend 905804 |
| Rabbit anti-CD45 | Immunofluorescence (1:100) | Abcam ab10558 |
| Rabbit anti-CD271 | Immunofluorescence and Immunocytochemistry (1:100) | Millipore 07-476 |
| Rabbit anti-phospho-PI3K | Immunocytochemistry (1:100) and Western Blot (1:1000) | Thermo Fischer PA5-104853 |
| Mouse anti-Cre | Immunocytochemistry (1:100) and Western Blot (1:1000) | Sigma-Aldrich MAB3120 |
| Mouse anti-CD271 | Western Blot (1:1000) | Millipore 05-446 |
| Rabbit anti-PI3K | Western Blot (1:1000) | Cell Signaling 4257 |
| Rabbit anti-AKT | Western Blot (1:1000) | Cell Signaling 9272 |
| Rabbit anti-phospho-AKT | Western Blot (1:1000) | Cell Signaling **4058** |
| Rabbit anti-ERK | Western Blot (1:1000) | Cell Signaling 9102 |
| Rabbit anti-phospho-ERK | Western Blot (1:1000) | Cell Signaling 4370 |
| Rabbit anti-K5 | Western Blot (1:1000) | BioLegend 905501 |
| Mouse anti-Involucrin | Western Blot (1:1000) | Sigma-Aldrich SAB4200794 |
| Rabbit anti-K10 | Immunofluorescence (1:100) and Western blot (1:1000) | BioLegend COD. 905404 |
| Rabbit anti-PKCα | Western blot (1:1000) | Cell Signaling 2056 |
| Rabbit anti-phospho-PKCα | Western blot (1:1000) | Life Technologies 44-962G |
| Rabbit anti-Claudin-1 | Immunofluorescence  (ready to use) | Abcam ab307692 |
| Mouse anti-CD68 | Immunohistochemistry  (ready to use) | Roche - Opti-View DAB immunohistochemistry automated detection kit (Ventana Medical Systems) |
| Mouse Anti-CD163 | Immunoistochemistry  (ready to use) | Roche Opti-View DAB immunohistochemistry automated detection kit (Ventana Medical Systems) |

Table S3: Primer sequence for Real-Time PCR

| *Ngfr* F | CAAGGGTGATGGCAACCTCT |
| --- | --- |
| *Ngfr* R | GCCTCGTGGGTAAAGGAGTC |
| *Dlx3* F | ATTACAGCGCTCCTCAGCAT |
| *Dlx3* R | CTTCCGGCTCCTCTTTCAC |
| *Filagrin* F | GAAACAGAAGACCCAGAGCAGACTCCCAGAAGG |
| *Filagrin* R | GACTGTCCTCTTCCTCCTGATCCCGATCTTGC |
| *Keratin* 10 F | TCACTGAATTGAGACGTACTGTTCAGGGTCTGG |
| *Keratin* 10 R | CTTGATGTCTAGGAGTTGTTGGTACTCGGCGTT |
| *P21-F* | GTG GCC TTG TCG CTG TCT T |
| *P21-R* | GCG CTT GGA GTG ATA GAA ATC TG |
| *IL1a -F* | CGA AGA CTA CAG TTC TGC CAT T |
| *IL1a-R* | GAC GTT TCA GAG GTT CTC AGA G |
| *Cxcl15-F* | GCT CTA GAA TGA CTT CCA AGC TGG CCG |
| *Cxcl15-R* | CGG GAT CCT TAT GAA TTC TCA GCC CTC T |
| *RPLPO-F* | ATCAATGGGTACAAGCGCGTC |
| *RPLPO-R* | CAGATGGATCAGCCAGGAAGG |

The TaqMan Array Applied Biosystem (Thermo Scientific) was used for the detection of Tgif and Pcna with the code Mm01227699_m1 and Mm05873628_g1, respectively.


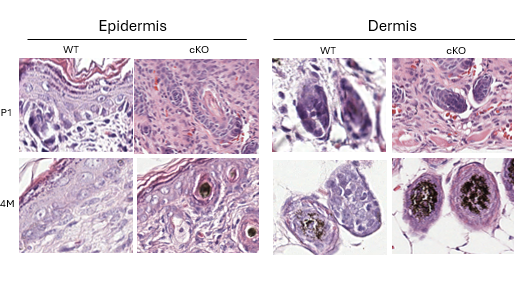


**Figure S1: CD271 deletion induces profound skin disorganization and increase number of hair follicles.** Detailed histological magnification of epidermis and dermis of cKO vs WT mouse skin at P1 and 4M of age.


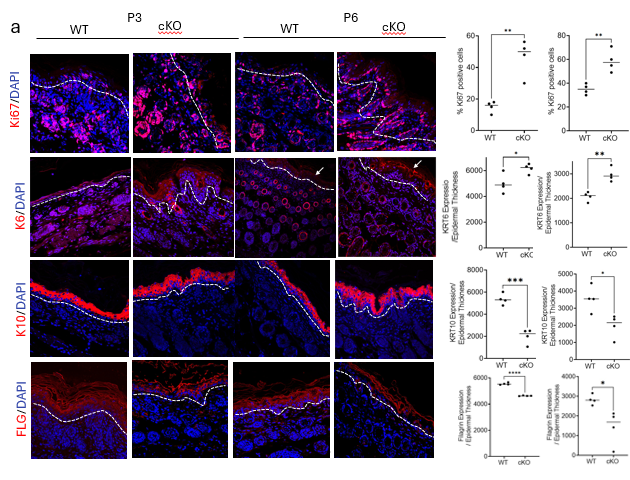


P3 P6


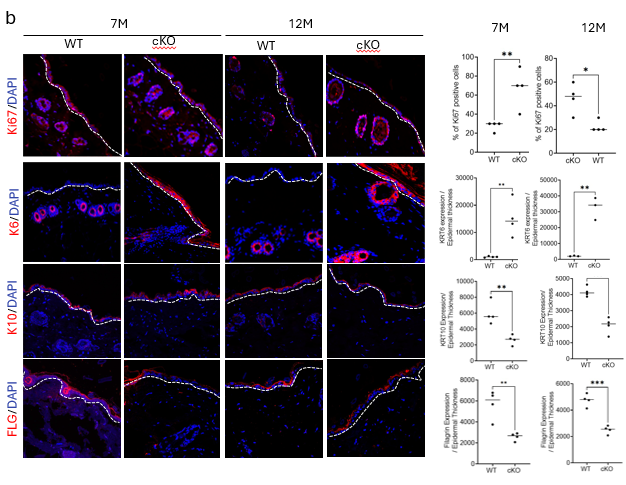


**Figure S2: CD271 deletion induces a dysregulation of epidermal proliferation and differentiation during mice development.** A-B) Representative images (Left panel) and relative quantification (Right panel) of the immunofluorescence staining for proliferation (Ki67 and KRT6) and differentiation (KRT1 and FLG) markers in cKO vs WT skin. Data are presented as mean ± SD from three independent experiments. Statistical analysis was performed using the T-test analysis. *: 0.01<p<0.05; **: 0.01<p<0.001; ***0.001<p<0.0001


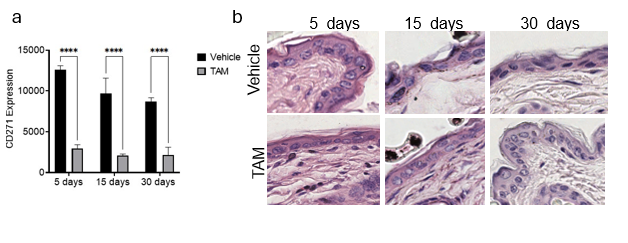


**Figure S3: Inducible CD271 deletion provokes skin disorganization in adult mice.** A) Fluorescence quantification of CD21 expression in TAM vs Vehicle-treated animals at 5, 15 and 30 dpt. B Histological detail of the epidermis in TAM vs Vehicle-treated animals at 5-, 15- and 30 dpt. Data are presented as mean ± SD from three independent experiments. Statistical analysis was performed using two-way ANOVA. ****p<0.0001


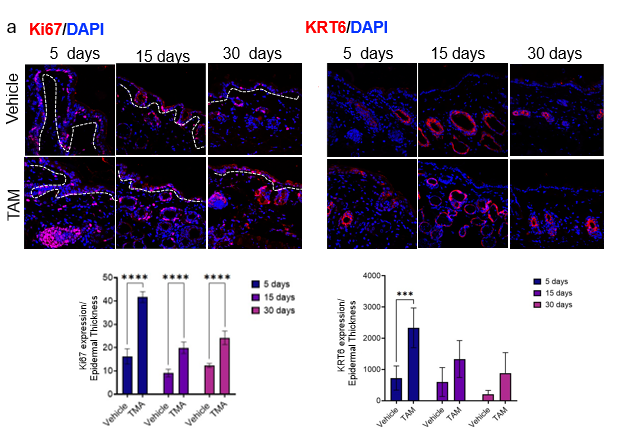


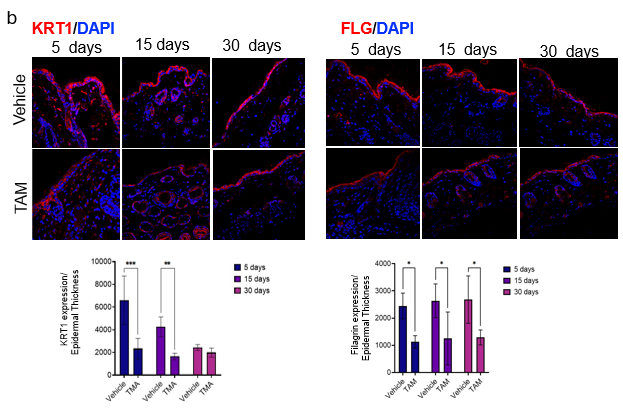


**Figure S4:** **Inducible CD271 deletion causes dysregulation of skin proliferation and disorganization in adult mice.** A) Representative image (Upper panel) and relative quantification (Below panel) of the immunofluorescence analysis of proliferation (Ki67 and KRT6) markers in TAM vs Vehicle treated animal at 5, 15 and 30 dpt. B) Representative image (Upper panel) and relative quantification (Below panel) of the immunofluorescence analysis of differentiation (KRT1 and FLG) markers in TAM vs Vehicle treated animal at 5, 15 and 30 dpt. Data are presented as mean ± SD from three independent experiments. Statistical analysis was performed using two-way ANOVA. *: 0.01<p<0.05; **: 0.01<p<0.001; ***0.001<p<0.0001; ****p<0.0001

**Figure S5: Inducible CD271 deletion causes dysregulation in hair follicles. A** Representative image of KRT6 in TAM vs. vehicle-treated animals at 5 and 30 dpt at the dermal level. **B** Western blot analysis (upper panel) of KRT6 expression in TAM vs. vehicle-treated animal with densitometric analysis (bottom panel). β-actin was used as reference protein. Data are presented as mean ± SD from three independent experiments. Statistical analysis was performed using two-way ANOVA. *****p* < 0.0001


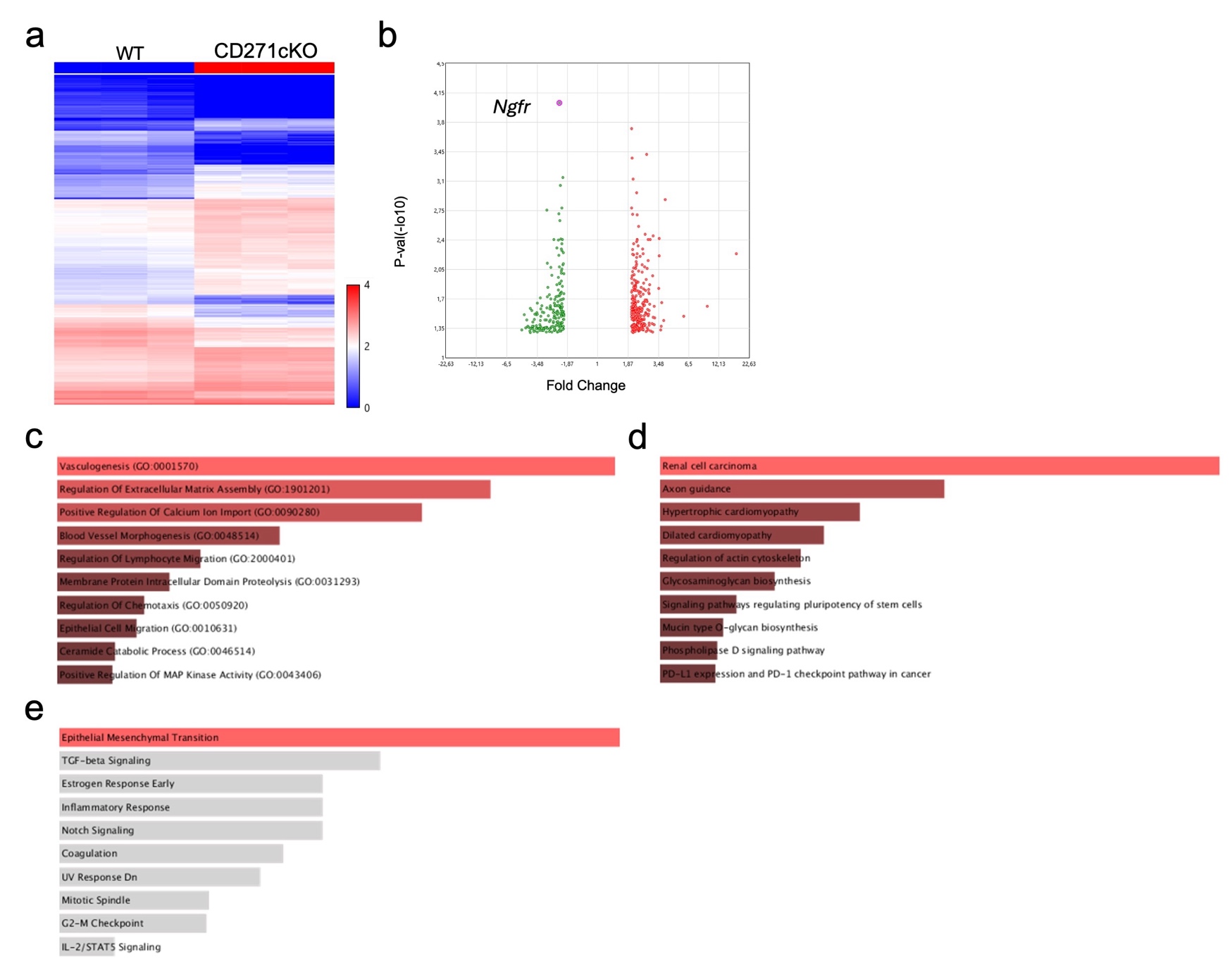


**Figure S6: CD271 loss modulates several skin regulatory pathways** A) Heatmap of the cKO vs WT skin DEG ranked according to the Log2 of fold change transformation (here referred as “Value”), obtained using the following formula: log2 (X+d), where “X” corresponds to fold change and “d” corresponds to a positive value greater than the lowest negative value. B) Volcano plot representation of upregulated (green) and downregulated (red) mRNA of cKO vs WT at P1; C-E) Pathway clustering analysis of the most significantly modulated genes in cKO vs WT P1 total skin performed by Enrichment WikiPathways 2024 Mouse, Enrichment KEGG 2021 Human and Enrichment Hallmark 2020, respectively.


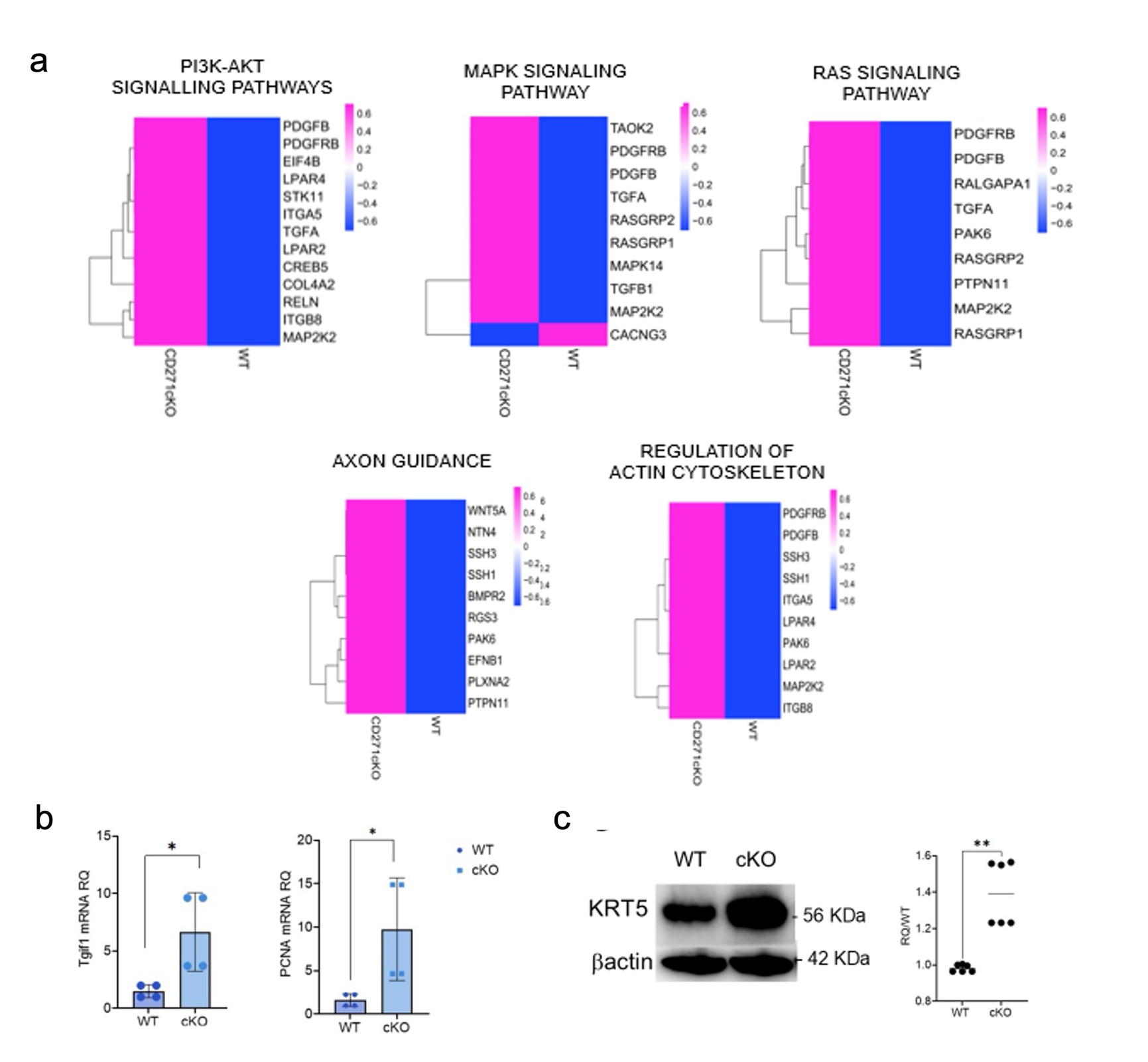


**Figure S7: CD271 KO provokes a profound modulation of the transcriptomic profile in mouse skin.** A) Heatmap of the most significant modulated genes in the top pathways cKO vs WT P1 total skin generated by SRPlot (Tang et al., PLoS One. 2023), clustered by row z-score. B) Evaluation of Tgif1 and PCNA mRNA expression by Real-Time PCR in cKO vs WT P1 skin. RPLPO was used as reference gene. C) Western blotting analysis and relative densitometric analysis of KRT5 expression in cKO vs WT P1 skin**.** β-actin was used as reference protein. Data are presented as mean ± SD from three independent experiments. Statistical analysis was performed using T-test or two-way ANOVA. *: 0.01<p<0.05; **: 0.01<p<0.001; ***0.001<p<0.0001; ****p<0.0001


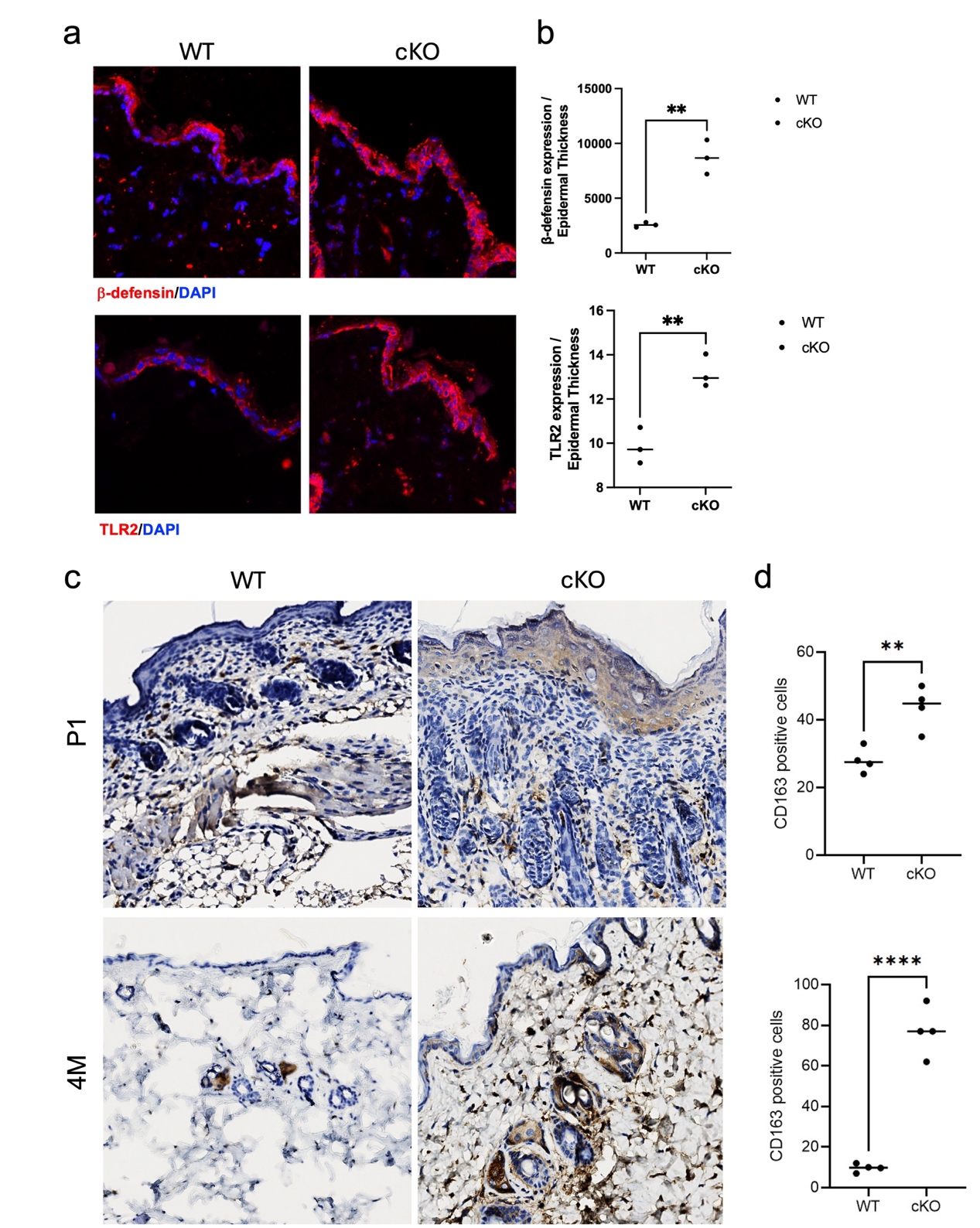


**Figure S8: CD271 deletion causes increased skin-barrier defects-related marker expression.** A-B Representative images (Left panel) and relative quantification (Right panel) of the immunofluorescence staining for β−defensin and TLR2 in cKO vs WT skin (Left panel). Data are presented as mean ± SD from three independent experiments. Statistical analysis was performed using the T-test. **0.001<*p* < 0.01

**
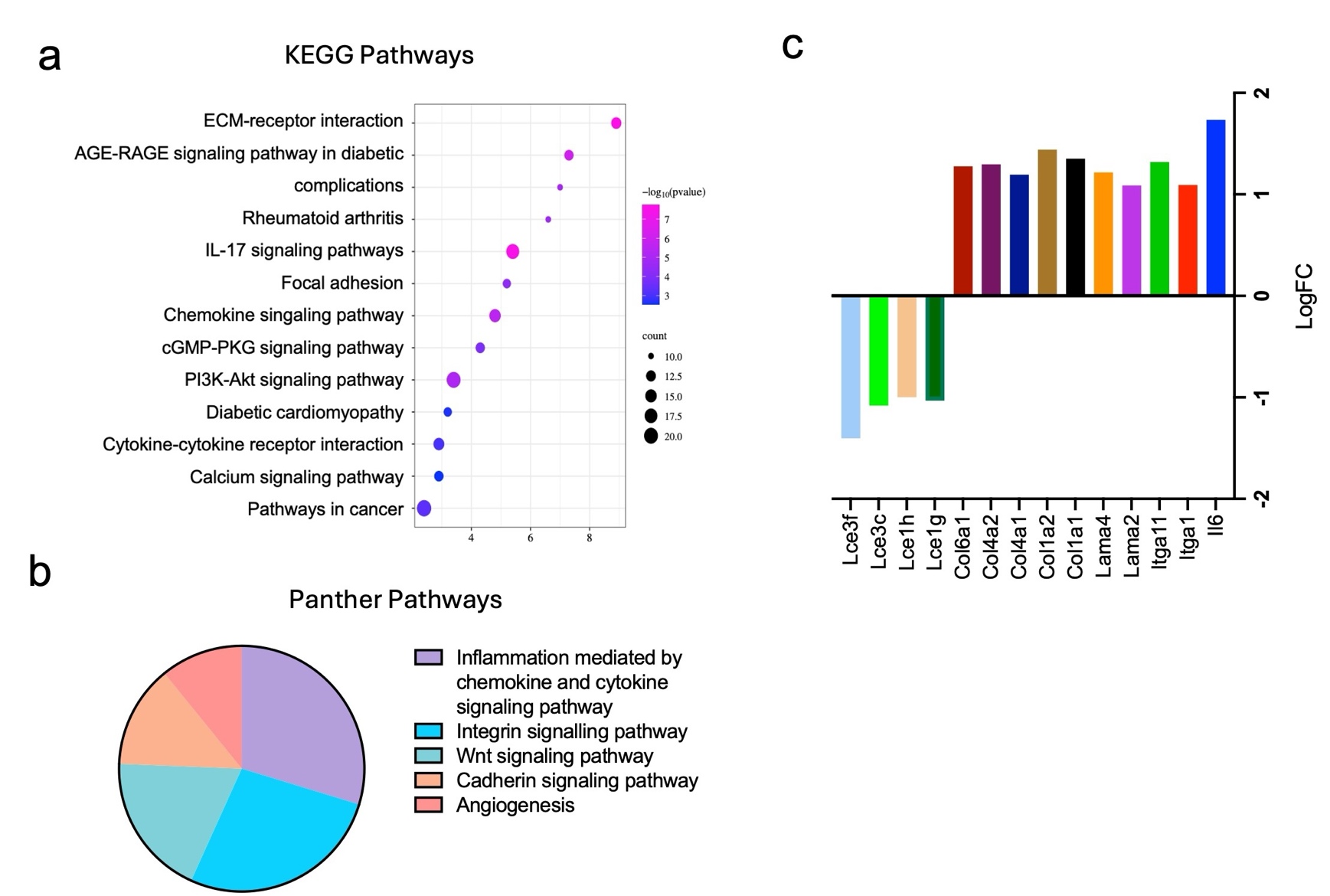
**

**Figure S9: Full CD271 loss leads to a profound alteration of the transcriptomic profile and modulates the activation of the PI3K/Akt pathways.** A**)** KEGG pathways, B) Panther pathways and C) skin barrier or ECM-related genes of the full CD271 KO vs WT dataset GSE9910 by Caporali et al., 2008.

**
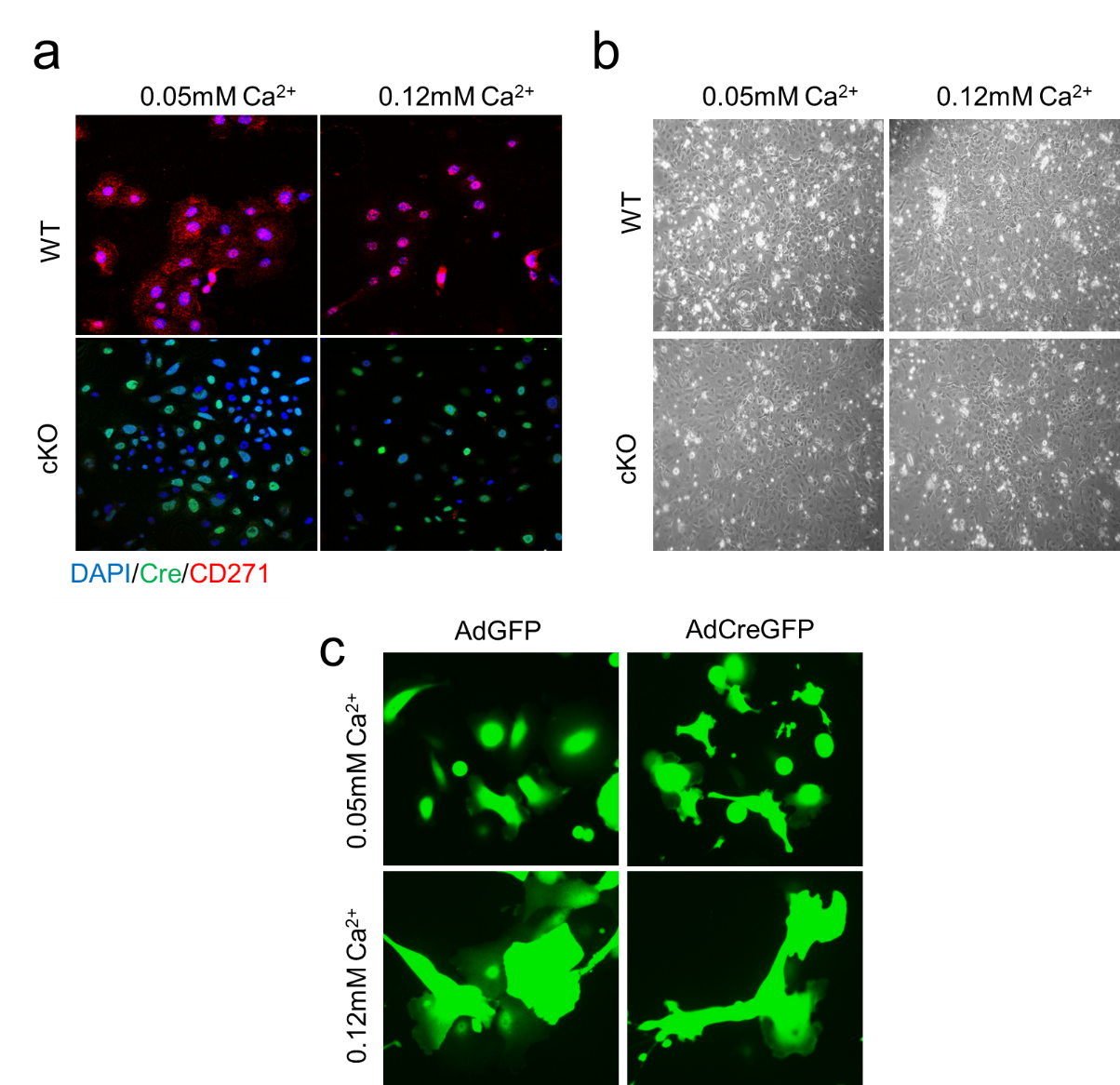
**

**Figure S10:** **CD271 knock-out in keratinocytes possess increased viability and resistance to differentiation stimuli by triggering mitogenic pathway activation. A** Representative image of the immunofluorescence staining for Cre (green) and CD271 (red) in WT and cKO PMK in Low Calcium (0.05mM Ca^2+^) and High Calcium (1.2mM Ca^2+^) conditions. **B** Representative images of cKO and WT cells in Low Calcium (0.05mM Ca^2+^) and High Calcium (1.2mM Ca^2+^) conditions. **C** Representative image of GFP expression in PMK cells treated with Adeno-GFP or Adeno-CreGFP viral vector in Low Calcium (0.05mM Ca^2+^) and High Calcium (1.2mM Ca^2+^) conditions.


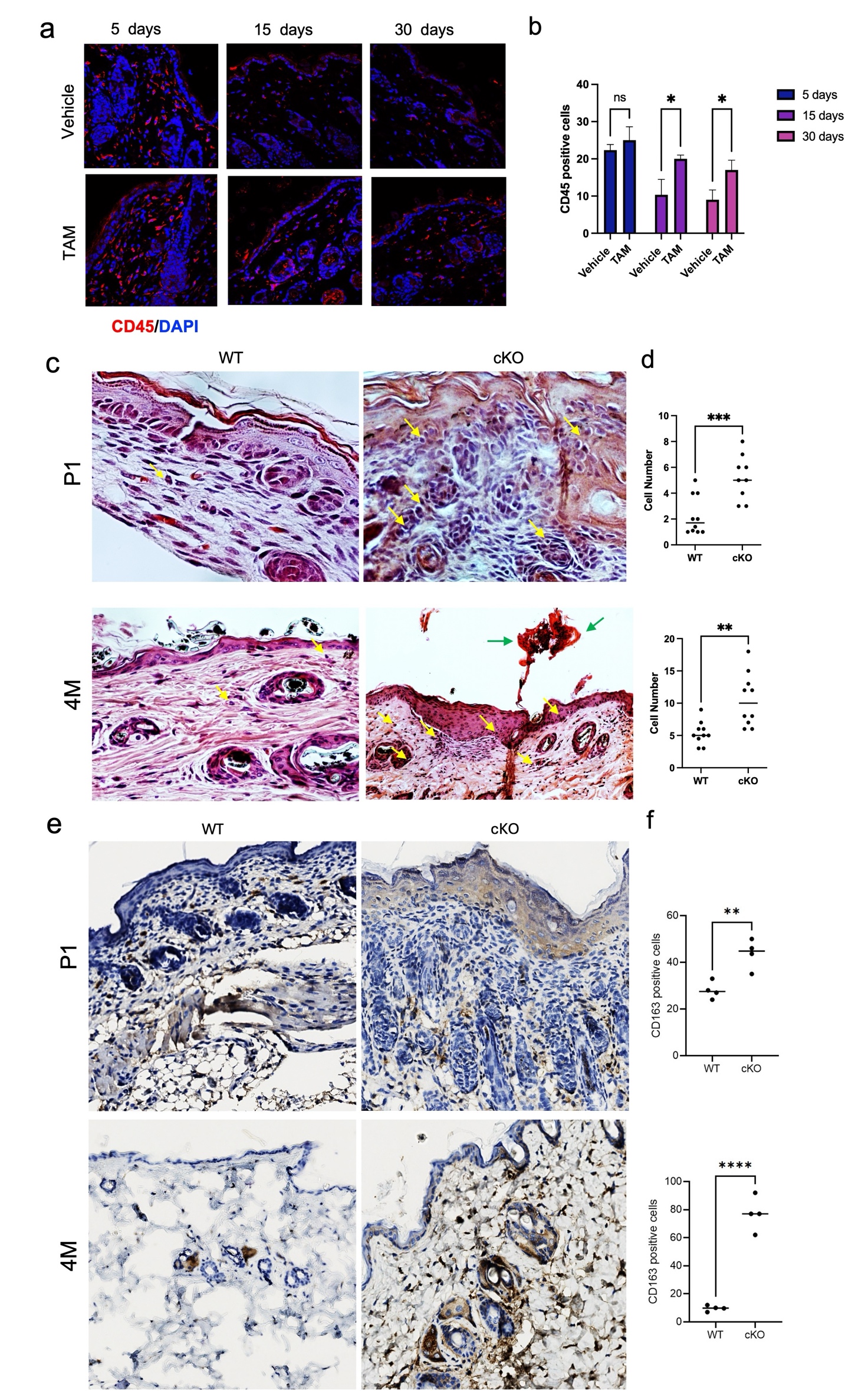


**Figure S11: CD271 deletion causes increased inflammation-related marker expression**. A) Representative image and B) relative quantification of the immunofluorescence staining of CD45 in TAM vs. vehicle-treated animals at 5, 15 and 30 dpt. C) Representative images and D) relative quantification of numbers of neutrophils in cKO versus WT mouse skin at P1 and 4M of age. E) Representative images and F) relative quantification of CD163 expression evaluated by IHC in cKO versus WT mouse skin at P1 and 4M of age. Data are presented as mean ± SD from three independent experiments. Statistical analysis was performed using the T-test analysis or two-way ANOVA. *: 0.01<p<0.05; **: 0.01<p<0.001; ***0.001<p<0.0001; ****p<0.0001
