# Supplementary material for: CD271 orchestrates skin structure, differentiation, and inflammation via PI3K/Akt and PKCα/ERK pathways
Source: Cell Death Dis. 2025 Oct 21;16(1):735. doi: 10.1038/s41419-025-08062-5 (PMC12540997; doi:10.1038/s41419-025-08062-5)

SUPPLEMENTARY MATERIALS 2

Originals Westen blot

Figure 1D

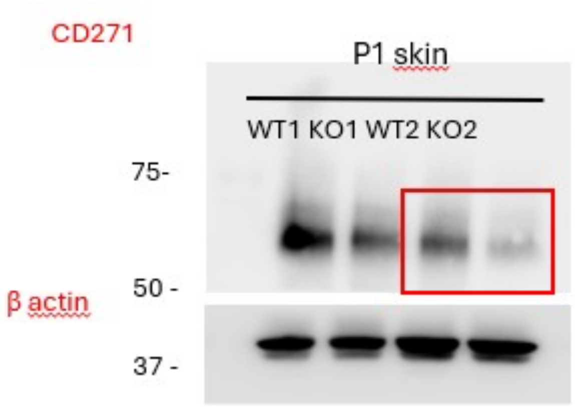

Figure 3D

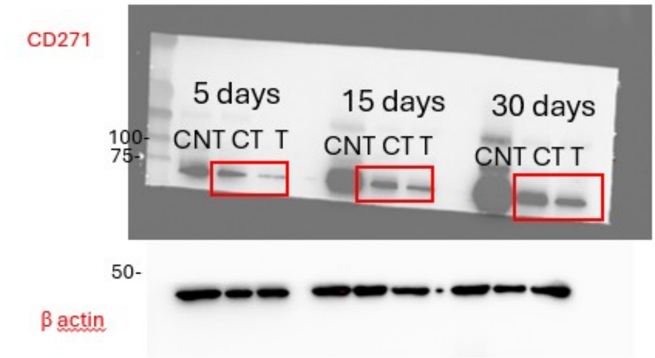

CNT: Skin non treted
CT: Skin treted with ethanol
T: Skin treated with Tamoxifen

Figure 5F

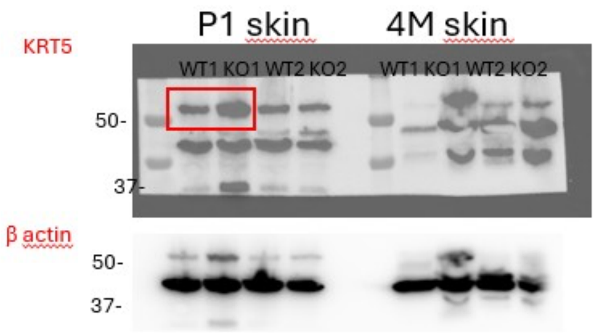

Figure 5K

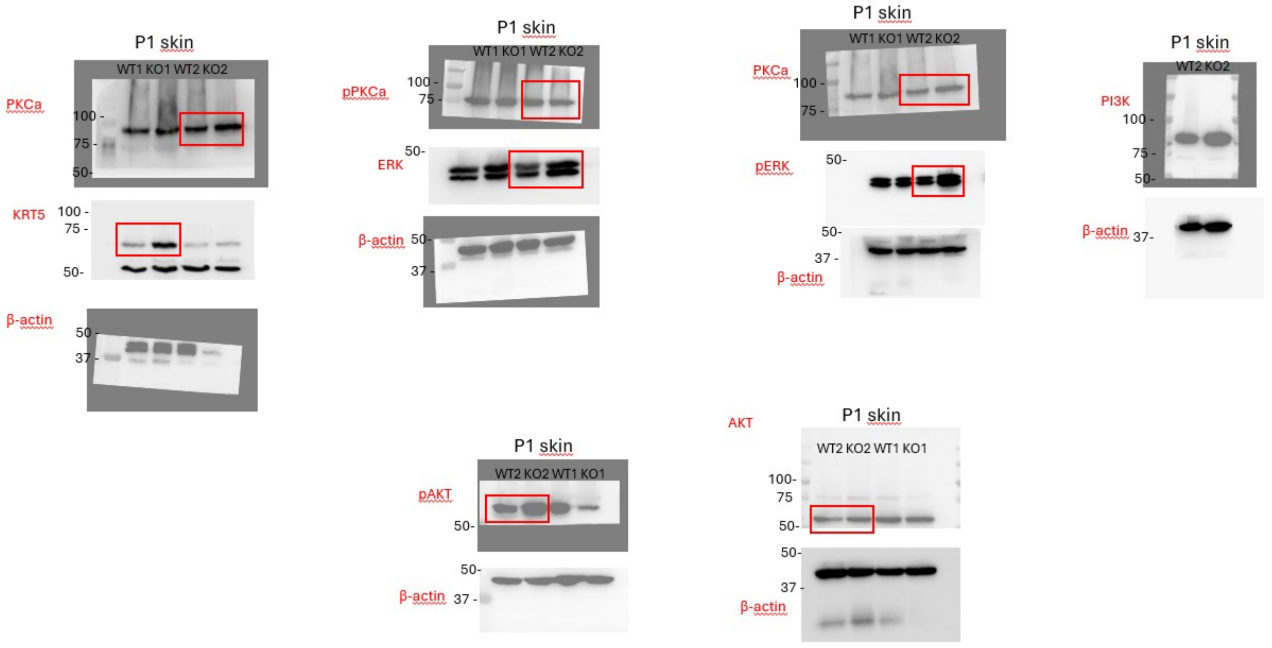

Figure 5L

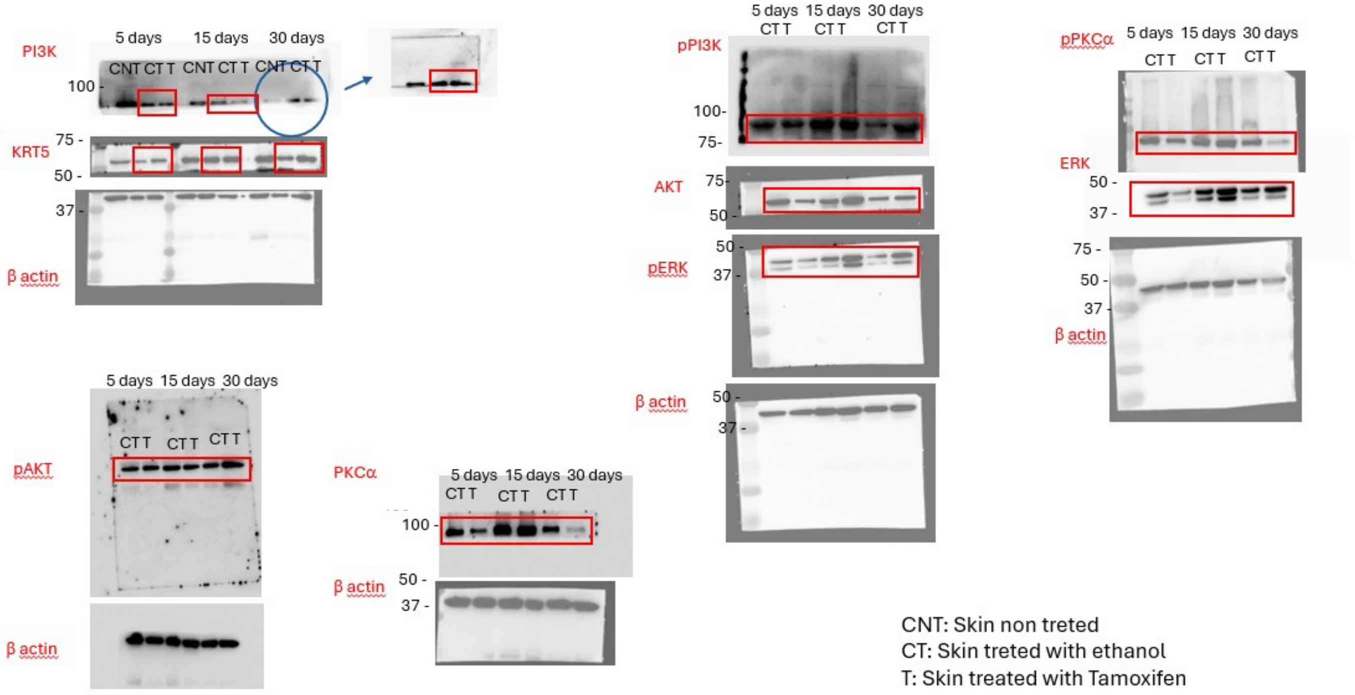

CNT: Skin non treted
CT: Skin treted with ethanol
T: Skin treated with Tamoxifen

Figure 6D

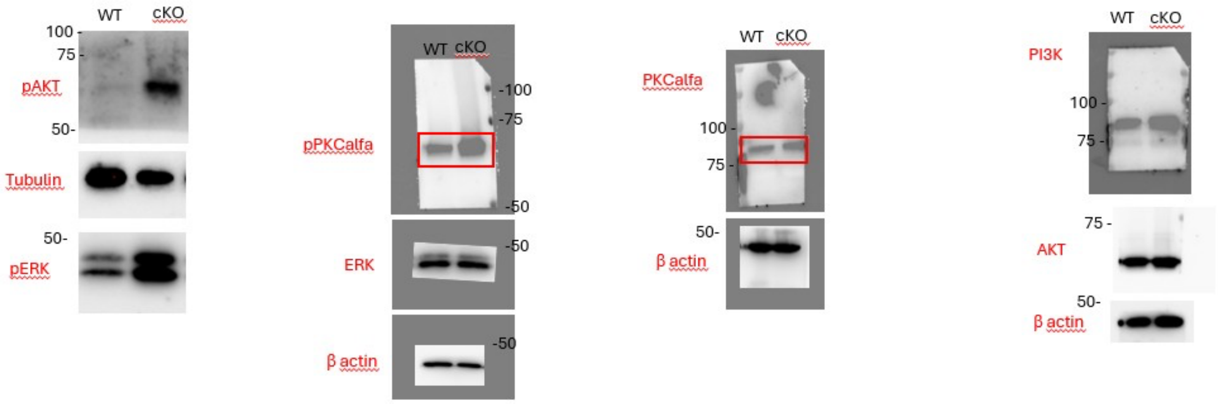

Figure 6E

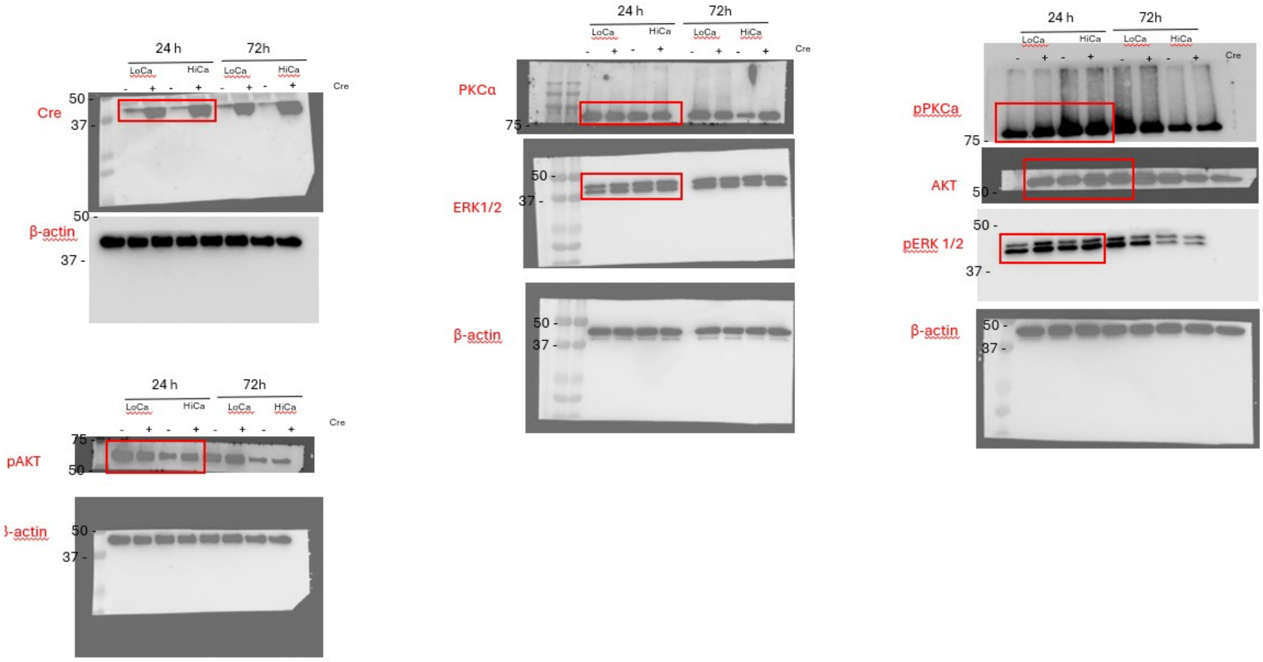

Figure 6G

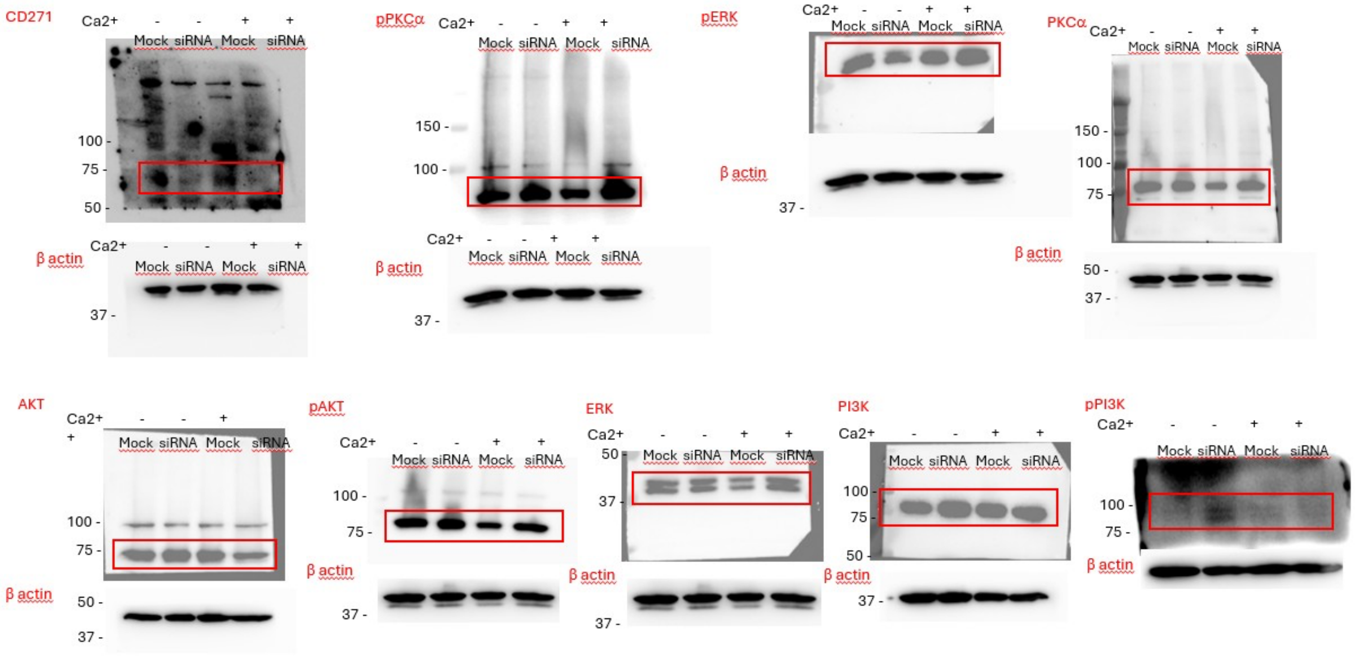

Figure S5

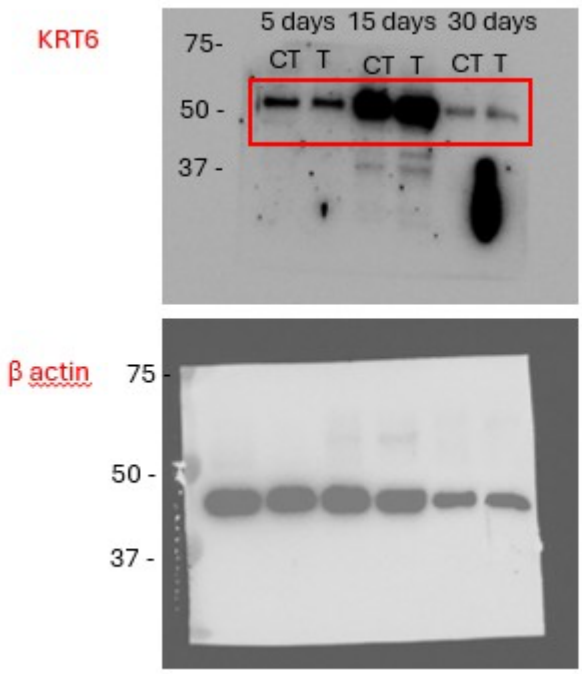

Supplement: Supplementary file 2 — Supplementary Materials [file 41419_2025_8062_MOESM2_ESM.pdf]
